# Automatic coarse-to-fine AC-PC localization on CT using registration-guided 3D-UNets

**DOI:** 10.3389/fninf.2026.1853412

**Published:** 2026-07-21

**Authors:** Sharada Kadaba Sridhar, Alyssa Eastman, Peter Wilson, Shubhendu Mishra, Charles Broadbent, Chip Truwit, Rui Kuang, Uzma Samadani

**Affiliations:** 1 Department of Bioinformatics and Computational Biology, University of Minnesota, Minneapolis, MN, United States; 2 Neurotrauma Research Lab, Center for Veterans Research and Education, Minneapolis, MN, United States; 3 Department of Radiation Oncology, University of Minnesota, Minneapolis, MN, United States; 4 Department of Computer Science and Engineering, University of Minnesota, Minneapolis, MN, United States; 5 Department of Radiology, University of Minnesota, Minneapolis, MN, United States; 6 Division of Neurosurgery, Department of Surgery, Minneapolis Veterans Affairs Health Care System, Minneapolis, MN, United States

**Keywords:** anterior commissure, computed tomography, deep learning, image registration, normal pressure hydrocephalus, posterior commissure

## Abstract

**Introduction:**

Automatically localizing the Anterior Commissure (AC) and Posterior Commissure (PC) is foundational for CT-based algorithmic disease screening, yet robust computational methods for this on CT remain lacking. We developed a registration-guided 3D-UNet framework for CT-based AC-PC localization, demonstrating its utility in computing ventriculomegaly features for Normal Pressure Hydrocephalus (NPH) detection.

**Methods:**

Framework development and evaluation were on an internal cohort of scans from patients with NPH, Alzheimer’s disease, post-traumatic volume loss, and headache (Veterans Affairs [VA]-Cohort, *n* = 427). External validation was on separate datasets (VA-ExtCohort, University of California, Santa Barbara [UCSB]-ExtCohort). AC-PC reference standard definition, model development, and evaluation were on 1 mm^3^–resampled scans.

**Results:**

On 1-mm^3^ resampled scans, test-set AC-PC mean radial errors (MREs) were 1.64/1.49 mm on the VA-Cohort, 2.42/1.79 mm on the VA-ExtCohort (*n* = 40), and 2.31/1.93 mm on the UCSB-ExtCohort (*n* = 43). Notably, the upper limits of the 95% confidence intervals (CIs) for localization errors across all cohorts were well below 3.2 mm; we empirically determined this to be a clinically relevant threshold beyond which the discriminative power of AC-PC-referenced ventriculomegaly features degrades. Ventriculomegaly features assessed using our framework’s predictions successfully distinguished NPH from Alzheimer’s disease, post-traumatic volume loss, and headache on a chart-verified VA-Cohort subset (*n* = 238) with a test-set Area Under the Receiver Operating Characteristic Curve (AUC) of 0.95, closely matching the performance of features assessed using manual AC-PC localization.

**Conclusion:**

The proposed registration-guided 3D-UNet framework accurately and automatically localizes the AC-PC on CT despite varied structural degeneration, enabling standardized radiological feature computation. This approach can augment neurodegenerative disease screening on CT, the primary modality for elderly patients evaluated for falls and altered mentation.

## 1 Introduction

Localization of the anterior (AC) and posterior (PC) commissures is critical to ensure reliability, precision, and standardized measurement of diagnostic-standard radiological markers of neurodegenerative diseases like Normal Pressure Hydrocephalus (NPH), Alzheimer’s disease, and chronic traumatic brain injury (Park and Moon, 2016; Lee et al., 2021; Ryska et al., 2021; Kadaba Sridhar et al., 2024a). Although the AC-PC localization problem on magnetic resonance imaging (MRI) has received sustained focus due to its high soft-tissue contrast and classical use in image-guided neurosurgery (Ardekani and Bachman, 2009; Liu and Dawant, 2015; Ghayoor et al., 2018), achieving accurate AC-PC localization on computed tomography (CT) is crucial. CT (with slice thicknesses up to 5 mm) provides density information for radiation dose calculations (Prabhakar et al., 2009) and aids in deep brain stimulation (Gubler et al., 2023), with utility poised to grow in robotic neurosurgery as scanner technology advances. CT is the primary modality to evaluate falls, which cause three million emergency visits annually (Centers for Disease Control and Prevention, 2026). It is quicker, more affordable, and less prone to geometric distortion (Khan and Gibbons, 2014), preferred for patients with implants, and more widely available in low-resource settings (O’Hanlon et al., 2020). Our analysis of the last decade’s records from a national Veterans Affairs (VA) database revealed that among patients with suspected dementia and unlikely intracranial injury, ~76% received CT only, ~10% MRI only, and ~14% both (Supplementary Table S1). This indicates a significant opportunity to leverage widely available yet underutilized CT imaging (Cauley et al., 2021) to algorithmically assess for neurological diseases that often underlie ataxia or instability precipitating falls (Stolze et al., 2004; Sennik et al., 2019). CT-based algorithmic AC-PC localization is additionally necessary given the incompatibility of alternative CT reference planes (also requiring manual localization) with AC-PC-referenced radiological markers (Yeoman et al., 1992; Kim et al., 2009).

Inferring AC-PC positions via standard registration-based solutions, such as fatbACPC (Rubbert et al., 2021), which remains the only dedicated tool for AC-PC alignment on brain CT, presents inherent limitations for accurately localizing these small white matter (WM) structures. Registration cost functions (normalized correlation or mutual information) depend on clear contrast boundaries between adjacent structures to learn optimal spatial mappings. In contrast, the internal brain parenchyma on CT lacks the distinct soft-tissue contrast needed to drive accurate local alignment (Yuan et al., 2021). Registration algorithms rely on iteratively matching spatial intensity distributions under constraints of limited degrees of freedom either through linear transforms or through regularization penalties in non-linear paradigms. These transformations fail to accurately map globally warped pathological anatomies onto normal templates (Ou et al., 2014) or require cohort-specific templates for improved registration (Pappas et al., 2021). These challenges may be mitigated by a CNN-based refinement of registration-based solutions for accurate AC-PC localization on CT scans, including those with ventricular distention in hydrocephalus and cortical volume loss in atrophy. Such a tool can enable standardized assessment of radiological markers not only for NPH, where 80% of cases are misdiagnosed as irreversible dementia (Kiefer and Unterberg, 2012), but also for population-level screening of neurodegenerative diseases characterized by the interplay of hydrocephalus and atrophy.

Inspired by deep learning approaches for MRI-based AC-PC localization (Edwards et al., 2021; Gohel et al., 2023), and coarse-to-fine localization strategies in low-contrast cephalometric landmark prediction (Zhong et al., 2019), we hypothesized that a deep learning framework informed by image registration can accurately localize the two commissures on CT. We developed a registration-guided 3D-UNet) framework for coarse-to-fine AC-PC localization on CT scans with varied structural degeneration. We evaluated the framework under 5-fold cross-validation on an internal dataset, using mean radial error (MRE), defined as the average Euclidean distance between the predicted and reference-standard (ground truth) AC-PC landmark predictions as the evaluation metric. We benchmarked its performance against the purely registration-based fatbACPC, a 3D-UNet baseline performing single-step AC-PC localization without the registration guide, and validated it on external datasets. We also analyzed its performance across different scan and clinical parameters. Importantly, we demonstrated the framework’s utility by applying its AC-PC predictions to standardize CT features for classifying potentially treatable NPH from its symptomatic mimics and by comparing its performance against features standardized using manually localized AC-PC.

## 2 Materials and methods

### 2.1 Overview

The methodology is organized as follows: study design and dataset creation (Section 2.2); image acquisition and preprocessing (Section 2.3); generation of the AC-PC reference standard for model training (Section 2.4); the proposed registration-guided 3D-UNet for coarse-to-fine localization (Section 2.5); benchmarking against a baseline 3D-UNet and an external registration-based AC-PC alignment tool (Section 2.6); and the NPH classification study evaluating the framework’s utility (Section 2.7). For multi-planar cohorts with all three CT reformats (axial, coronal, and sagittal) available, we developed reformat-specific models that share the same architecture (both registration-guided and baseline 3D-UNets) and use identical training and hyperparameter tuning strategies. Final predicted AC-PC coordinates were derived by integrating the reformat-specific predictions: x-coordinate averaged from axial and coronal model predictions, y-coordinate from axial and sagittal model predictions, and z-coordinate from coronal and sagittal model predictions. This approach excluded the sub-optimally resolved slice dimension of each reformat. We utilized this integrated framework because routine CT natively supports multi-planar reformats, the reformat-specific predictions are parallelizable. It aligns with the multi-planar nature of our downstream NPH classification analysis. We also evaluated the proposed model’s robustness for AC-PC localization on an axial reformat-only dataset.

### 2.2 Study design and datasets

**Figure 1:**
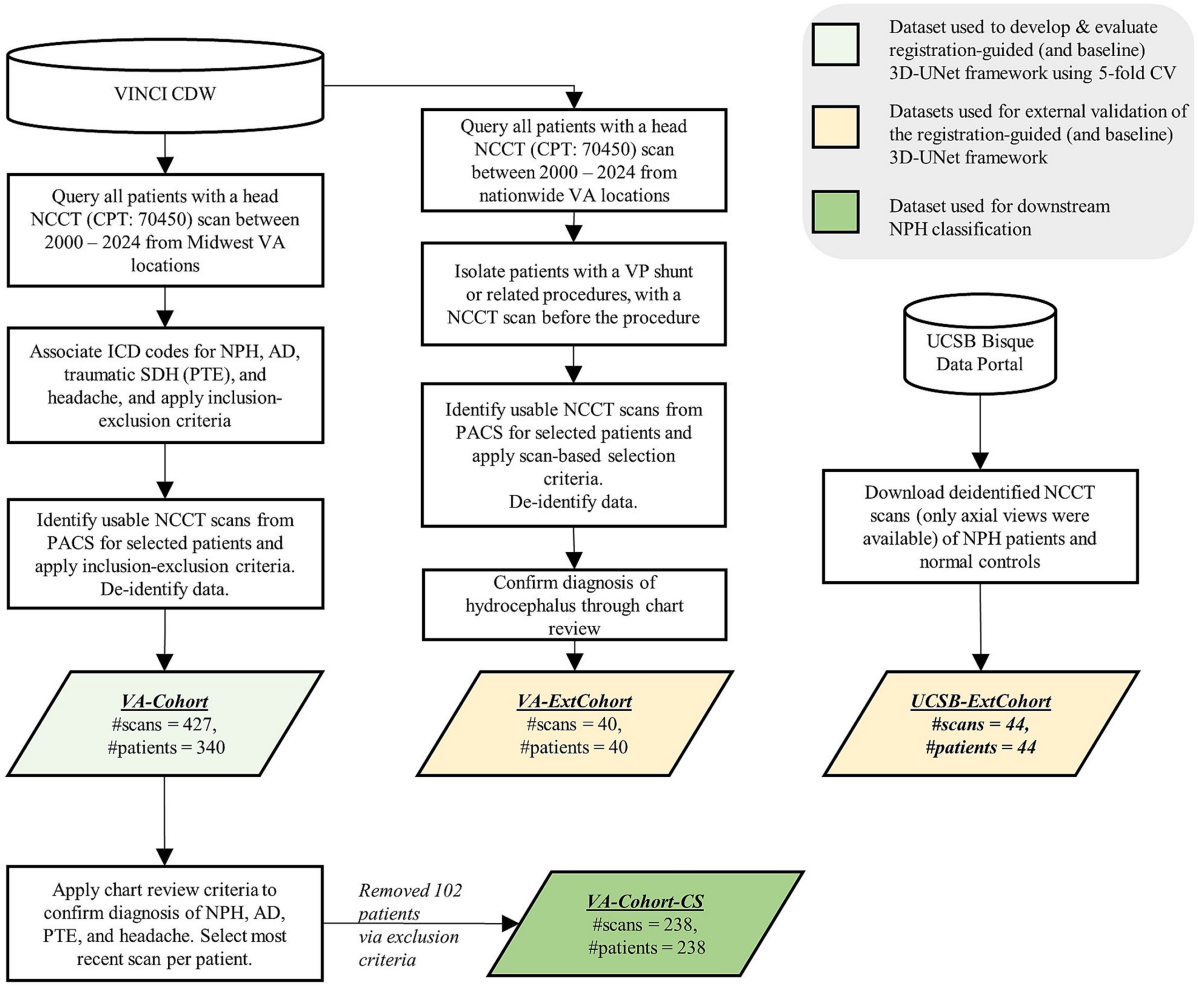
Study dataset creation. An overview of the acquisition processes for the study’s datasets, along with the analyses they were used for, is provided. Note that the VA-Cohort-CS is the chart review-qualified subset of the VA-Cohort, used for the NPH classification study. VA, Veterans Affairs; CPT, Current Procedural Terminology; ICD, International Classification of Diseases; NPH, Normal Pressure Hydrocephalus; AD, Alzheimer’s disease; SDH, subdural hemorrhage; PTVL, Post-traumatic volume loss; VP, Ventriculoperitoneal; PACS, Picture Archiving and Communication System; and UCSB, University of California, Santa Barbara, CV, cross-validation.

Figure 1 summarizes the dataset creation process and the specific analyses each cohort supported. In total, this study utilized three Institutional Review Board-approved datasets with informed consent waivers: two retrospective cohorts from the Veterans Affairs (VA) healthcare system (spanning 2000–2024; see Supplementary Table S2 for inclusion–exclusion criteria), and a third dataset provided by Zhang et al. (2022) from the University of California, Santa Barbara (UCSB). Age was available for all cohorts; biological sex was collected for the VA cohorts but was unavailable for the UCSB cohort.

- VA-Cohort (Internal dataset; 427 scans from 340 patients; multi-planar CT): This dataset consisted of patients with suspected NPH (pre-op scans), Alzheimer’s disease, post-traumatic volume loss (volume loss following a traumatic chronic subdural hemorrhage with near-to-complete lesion resolution), and headache (utilized as a control proxy). We used this dataset, which includes diverse structural pathology, to develop and test the proposed AC-PC localization framework.
- VA-Cohort-CS (NPH classification dataset; *n* = 238 scans [1 per patient]; multi-planar CT): A chart-review-qualified subset of the VA-Cohort containing the single most recent scan from patients with confirmed neurological conditions (NPH [pre-op scans], Alzheimer’s disease, post-traumatic volume loss, and headache). This specific subset, which was studied in our prior research to classify NPH via CT features derived using manual AC-PC localization (Kadaba Sridhar et al., 2024b). It was utilized in the current study to demonstrate the proposed automated framework’s utility in replacing manual AC-PC localization for downstream NPH classification.
- VA-ExtCohort (Test-only; *n* = 40 scans [1 per patient]; multi-planar CT): Pre-op scans from VA patients with ventricular shunting for hydrocephalus due to various etiologies (e.g., hemorrhage, tumor, aqueductal stenosis, and meningitis). Although this cohort represents diverse underlying causes, we excluded patients whose scans demonstrated intracranial lesions or acute findings (including significant midline shift).
- UCSB-ExtCohort (Test-only; *n* = 44 scans [1 per patient]; axial-only CT): NPH patients (pre-op scans) and normal subjects.

### 2.3 Image acquisition and preprocessing

For the VA-Cohort, the median slice thickness and axial in-plane resolution were 5 and 0.47 mm, respectively. In the VA-ExtCohort, these values were 3 and 0.47 mm, while the UCSB-ExtCohort had a thickness of 5 mm and an in-plane resolution of 0.49 mm. Supplementary Note 1 provides additional details on image acquisition. All scans underwent automated gantry tilt correction, isotropic resampling with cubic B-splines, cropping, bed and skull removal, and windowing (L/W: 50/100 Hounsfield units), using 3D-Slicer (Fedorov et al., 2012). Methodology development and validation, including obtaining the AC-PC reference standard, were performed on isotopically resampled 1-mm^3^ scans. Although this resampling does not add new information, it was pragmatically necessary to (1) enhance perceived resolution for the generation of a precise manual reference standard, (2) improve spatial correspondence to the chosen 1mm^3^ template used for registration (coarse localization) (Zhao et al., 2016), and (3) standardize the physical dimensions of image patches input to 3D-UNets. A standard skull-stripping approach (Rajashekar et al., 2020), with empirically derived parameters (2-mm Gaussian smoothing, 3-mm erosion/dilation), was used for robust skull-stripping and to facilitate registration with the skull-stripped CT template. Compared to a more relaxed paradigm (such as 4 mm smoothing and 1.5-mm kernels), these strict parameters effectively eliminated residual bone, substantially improving subsequent registration to the skull-stripped CT template. All scans successfully passed this automated pipeline with a 0% failure rate, as confirmed by visual inspection.

### 2.4 Generating reference-standard AC-PC annotations

AC-PC coordinates were annotated along the central intercommissural line (Choi et al., 2013) using 3D-Slicer, following a protocol guided by a board-certified neuroradiologist with 34 years of experience (CT). SKS. (PhD candidate in Computational Biology) served as the Primary Annotator (PA) and annotated the VA-Cohort after training with CT. We obtained a second set of annotations on the VA-Cohort from three trained secondary annotators (SAs): two radiation-oncology residents (SM as SA1; PW as SA2) and a postgraduate healthcare scientist (AE as SA3), all of whom were blinded to PA annotations. SAs 1–3 annotated randomly sampled, mutually exclusive subsets of the VA-Cohort to provide a complete set of secondary annotations on the data.

A trained PhD candidate in Computer Science (CB as SA4) annotated the VA-ExtCohort, and the PA annotated the UCSB-ExtCohort. All annotations were systematically audited by uninvolved annotators to verify adherence to the standard protocol and finalized through a consensus workflow. See Supplementary Note 2 and Supplementary Figure S1 for details on annotation creation and workflow. Finalized PA annotations served as the reference standard for model development, SA1–3 annotations were used to assess inter-annotator agreement, and SA4 and PA annotations were used for validation on the VA-ExtCohort and UCSB-ExtCohort, respectively.

### 2.5 Registration-guided 3D-UNet for AC-PC prediction

**Figure 2:**
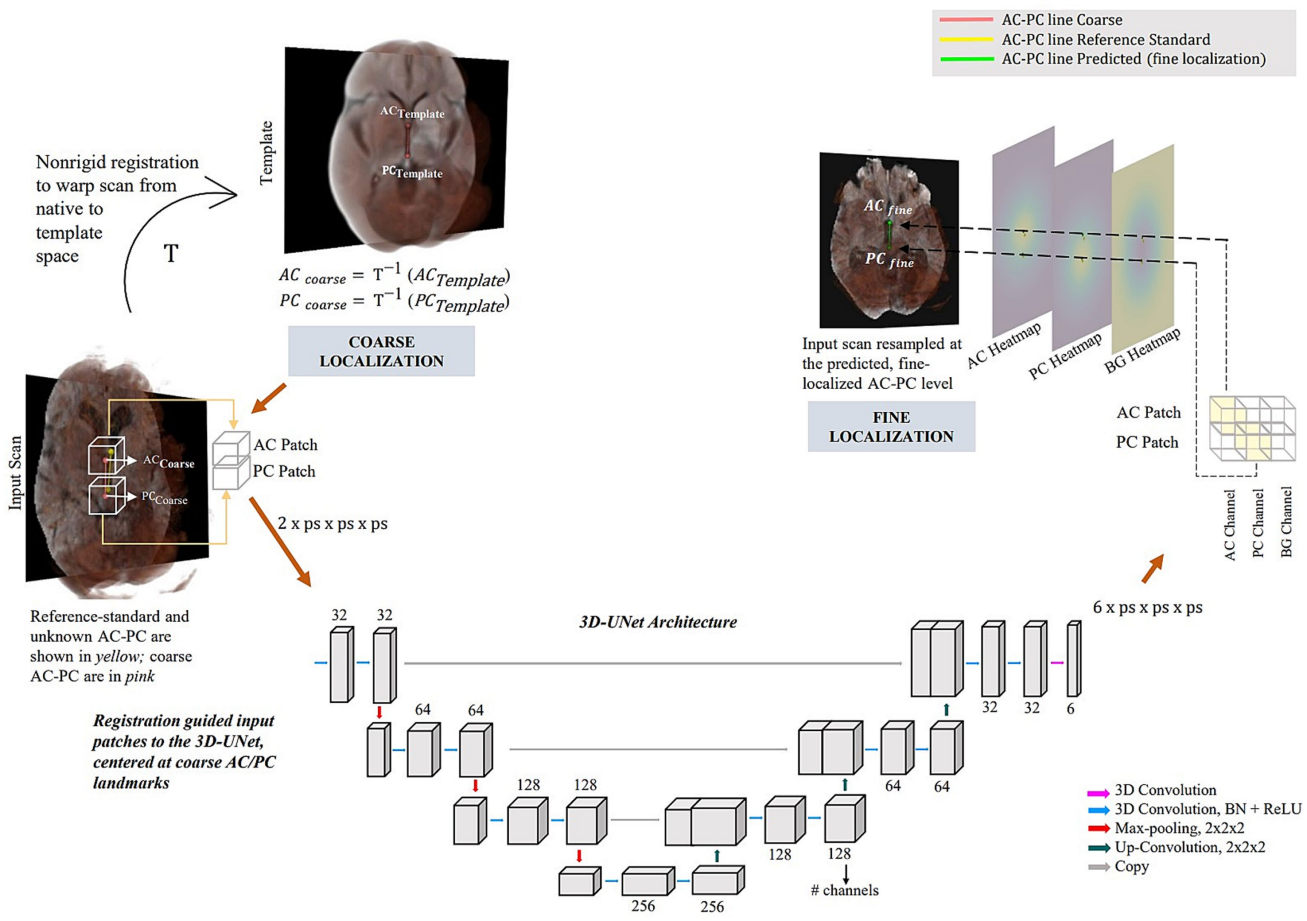
Coarse-to-fine AC-PC localization by the registration-guided 3D-UNet. Coarse landmark predictions inferred via image registration (coarse localization) guide the selection of image patches, which are input to the 3D-UNet to perform heatmap regression around the true AC and PC landmark predictions, along with a background heatmap. Patch- and channel-specific predictions are unstacked and normalized, followed by derivation of AC-PC predictions (fine localization) as the mean location of active voxels in the regressed heatmaps. AC, Anterior Commissure; PC, Posterior Commissure; BG, Background; ps, patch size; BN, Batch Normalization; ReLU, Rectified Linear Unit. Note that processing is in 3D, and 2D input slices and heatmaps are indicated for demonstration purposes.

Figure 2 depicts the registration-guided 3D-UNet for AC-PC localization. Coarse localization was first achieved as follows. Each scan was non-linearly registered to the CT template (Rajashekar et al., 2020) using 3D Slicer’s Elastix toolbox (Klein et al., 2010), after they were center-aligned with the template. We retained Slicer-Elastix’s default settings: a non-rigid registration transformation ( T ), parameterized with B-splines, was derived by minimizing the discrepancy between the fixed and transformed moving images using mutual information as the cost function; adaptive stochastic gradient descent optimized the cost function in a multi-resolution scheme. Next, known AC and PC landmark predictions on the central intercommissural line were identified on the template and passed through $\;T^{-1}$ , followed by the inverse of the center alignment transformations to obtain coarse localizations of the landmark predictions in the native image spaces. See Supplementary Figure S2 for a visual illustration of the process. Because this initial stage relies exclusively on standard atlas-based registration to obtain coarse AC-PC estimates (prior to any deep learning refinement), its performance also serves as a baseline for pure non-linear registration.

To perform fine localization of the landmark predictions via supervised heatmap regression, we modified the standard 3D U-Net (Çiçek et al., 2016) (architecture detailed in Figure 2). The network took two-channel inputs comprising 32 mm isotropic 3D patches cropped directly around the initial coarse AC and PC estimates. The model was trained to predict three confidence heatmaps per input channel, corresponding to the AC, PC, and background. Consequently, the ground-truth labels comprised six channels: isotropic 3D Gaussian heatmaps (standard deviation [SD], *σ*) centered at the AC and PC reference standards, alongside a normalizing background heatmap, all cropped to correspond to input patches centered at the coarse AC and PC. Mini-batch gradient descent with momentum minimized the loss function defined in Equation 1, which combined a focal loss (Lin et al., 2017) to learn the dense foreground (AC-PC landmark predictions) with cross-entropy to learn the over-represented background.

$$L=\frac{1}{|b|}*\sum_{i=1}^{|b|}{\left\{{\left(-\frac{1}{2}H\;\log(\hat{H})-\frac{1}{2}\alpha_{t}{\left(1-H_{t}\right)}^{\gamma }\log(\;H_{t})\right)}_{v_{\mathrm{avg}}}\right\}}^{\left(i\right)}$$ (1)

$\hat{H}$ and H denote the predicted and ground-truth heatmaps, respectively. $H_{t}$ was defined as:

$$H_{t}=\{\begin{array}{c} \hat{H}\;\text{if}\;H\geq \tau \\ 1-\hat{H}\;\text{elsewhere} \end{array}$$ (2)

$\alpha_{t}$ was defined similarly to $H_{t}$ in Equation 2, where *α* is the class imbalance parameter. τ is the minimum threshold to pick active voxels from heatmaps (set to 0.01), 𝛾 the focus parameter, $v_{\mathrm{avg}}\;$ indicates averaging heatmaps over all voxels in H , and i indexes over samples in the mini-batch b. Final AC-PC predictions for a given reformat were inferred by normalizing AC and PC patches from the corresponding output heatmaps, then calculating the mean location of active voxels within each patch. Reformat-specific registration-guided 3D-UNets were identically derived using this methodology. For multi-planar datasets (VA-Cohort, which was used for training and 5-fold cross-validation, and the VA-ExtCohort, which was used for external validation), the final AC-PC prediction framework simply integrated predictions from these reformat-specific models as described in Section 2.1.

### 2.6 Baseline 3D-UNet and external benchmark comparisons

We compared the localization performance of the proposed registration-guided 3D-UNet against a baseline 3D-UNet that localized AC-PC across whole scans without registration guidance. For computational feasibility, the scans were resized to 128 × 128 × 128 (single-channel input). The baseline model predicted heatmaps for the AC, PC, and a normalizing background (three-channel output), with final AC-PC predictions inferred as the mean location of active voxels within their corresponding channels. The model used the same preprocessing, loss function, optimizer, and hyperparameters as the registration-guided 3D-UNet. This methodology was applied identically to all three reformats to derive reformat-specific baseline 3D-UNets. As with the registration-guided 3D-UNet framework, the final baseline framework integrated AC and PC predictions from these reformat-specific models for multi-planar datasets.

We also benchmarked our framework against inferred AC-PC localization using fatbACPC, an external, purely registration-based tool for the AC-PC alignment of head CTs (Rubbert et al., 2021). Note that three scans (2 out of 427 in the internal VA-Cohort and 1 out of 43 in the UCSB-ExtCohort) were excluded from this comparison due to the lack of original Digital Imaging and Communications in Medicine (DICOM) files required by fatbACPC. Attempts to bypass this limitation by providing files in the Neuroimaging Informatics Technology Initiative (NIfTI) format resulted in invalid (negative) voxel coordinates, indicating an alignment failure within the tool. For the remaining scans, as per the module’s specifications, unprocessed native axial-reformatted CTs were passed into fatbACPC, which initially crops the neck and lower head while retaining the skull for registration. The tool outputs a rigid 6-DOF transformation that approximates the affine transformation mapping native scans onto an AC-PC-aligned template. Landmark predictions were derived using the same paradigm as our coarse localization step: we inverted the rigid subject-to-atlas transformation matrix produced by fatbACPC. We mapped known AC and PC atlas coordinates into the native scan space.

### 2.7 Evaluating the proposed framework’s utility in eliminating manual AC-PC localization bottleneck for NPH classification

To evaluate our framework’s clinical utility, we classified NPH from its symptomatic mimics (Alzheimer’s disease and post-traumatic volume loss) and headache patients in the VA-Cohort-CS using ventriculomegaly features anchored by the framework’s AC-PC predictions. We previously demonstrated that eight semi-automatically derived ventriculomegaly features, including seven AC-PC-dependent features and the AC-PC-independent cerebrospinal fluid to brain volume ratio, differentiate NPH from Alzheimer’s disease, post-traumatic volume loss, and headache with high accuracy when computed using manually localized AC-PC landmark predictions (Kadaba Sridhar et al., 2024b) (see Supplementary Figure S3 for explicit feature definitions). To evaluate the feasibility of replacing this manual AC-PC localization with our automated framework, we first established a clinical precision benchmark via a perturbation study. The seven AC-PC-dependent features were recomputed using AC-PC coordinates artificially perturbed from the reference standard with increasing noise (0 to 8 mm mean radial errors, in 1.6 mm increments), yielding Features-PerturbACPC. The noise threshold that significantly diminished multivariate logistic regression (LR) classification performance across binary and multi-class settings established the baseline precision required for clinical utility (Supplementary Note 3).

Next, we trained multivariate LR models under the same classification settings, using the seven AC-PC-dependent features derived directly from our framework’s predictions (Features-PredACPC). Their performance was compared with models trained on the manually annotated/ground-truth AC-PC-referenced features (Features-RefACPC) using McNemar’s tests. All classification models were evaluated using the same patient-level 5-fold cross-validation splits applied to the AC-PC prediction framework, ensuring a robust end-to-end evaluation (Supplementary Figure S4).

Python 3.12 (Python Software Foundation, 2023) and 3D-Slicer were used for methodology development and analysis (Sections 2.3–2.7). Significance for statistical tests was set at *p* < 0.05 (Benjamini–Hochberg-corrected wherever necessary).

## 3 Results

### 3.1 Dataset characteristics

Mann–Whitney *U*-tests were used to compare age and absolute head tilt distributions between the VA-Cohort (development/testing) and the external validation cohorts (VA-ExtCohort, UCSB-ExtCohort). Regarding the final dataset characteristics (Table 1), although the median ages were similar, the VA-ExtCohort was significantly younger than the VA-Cohort (median age 70 vs. 73 years; *U* = 5,172, *p* = 0.01), reflecting a leftward shift. The UCSB-ExtCohort showed a comparable age range with no significant difference from the VA-Cohort (median age 74 vs. 72 years; *U* = 7,178, *p* = 0.85). Less than 7% of the VA cohorts were female, a skew attributable to the male-dominated demographics of the aging veteran population affected by the neurodegenerative conditions being studied. Sex information for the UCSB-ExtCohort was unavailable. Absolute head-tilts were calculated using the AC-PC reference standard coordinates, where θ_yz_ = 0° corresponds to AC_z_ = PC_z_ and θ_xy_ = 0° corresponds to AC_x_ = PC_x_. They were comparable between the VA-ExtCohort and the VA-Cohort (θ_yz_: 9.2° vs. 7.6°, U = 8,897, *p* = 0.66; θ_xy_: 2.7° vs. 2.8°, *U* = 8,491, *p* = 0.95) but significantly higher in the UCSB-ExtCohort vs. the VA-Cohort (θ_yz_: 14.4° vs. 7.6°, *U* = 11,863, *p* = 0.002; θ_xy_: 6.3° vs. 2.8°, *U* = 12,647, *p* < 0.001). One UCSB-Cohort scan (θ_yz_ > 40^°^) failed the coarse-localization step, leaving a final sample of 43 patients.

**Table 1:** Dataset characteristics.

Dataset	Number of patients	Number of scans	Age (median [IQR])	Number of females
VA-Cohort				
AD	108	169	83 [78–88]	3
Headache patients	79	80	58 [53–68]	16
NPH	89	114	78 [73–83]	1
PTVL	64	64	73 [63–83]	1
*Total*	340	427	73 [68–83]	21
VA-ExtCohort				
Hydrocephalus	40	40	70 [65–75]	3
UCSB-ExtCohort*				
NPH	13	13	83 [80–85]	–
NC	30	30	71 [66–77]	–
*Total*	43	43	74 [67–82]	NA
VA-Cohort-CS^#^				
AD	62	62	83 [78–88]	1
Headache patients	59	59	58 [53–68]	14
NPH	64	64	78 [73–83]	1
PTVL	53	53	73 [63–83]	1
*Total*	238	238	73 [63–83]	17

Age (in years) is given as the median and the interquartile range (IQR).

AD, Alzheimer’s disease; NPH, Normal Pressure Hydrocephalus; PTVL, Post-traumatic Volume loss; NC, Normal Controls; VA, Veterans Affairs; UCSB, University of California, Santa Barbara; NA, Not Available. *UCSB-ExtCohort had only axial CT available, and VA-Cohort and VA-ExtCohort had all three multi-planar reformats for each scan. ^#^VA-Cohort-CS represents a subset of VA-Cohort, identified through chart review for diagnoses of NPH/AD/PTVL/headache patients, and was used in the NPH-Classification study.

A consistent and precise AC-PC reference standard was verified as follows. For the VA-Cohort AC-PC annotations between the PA and SAs (1–3) averaged within 1 mm of each other, the 75th percentile and maximum AC-PC radial errors (REs) were 1.22/1.25 and 3.84/3.35 mm, respectively (Supplementary Table S3). On 98.5% of scans, AC and PC annotations by the PAs and SAs each agreed within 3.2 mm. An extended Bland–Altman (BA) plot (Möller et al., 2021) on a common set of 36 scans showed no annotator bias, and agreement limits within 1.1 mm across all AC-PC coordinates (*x*/*y*/*z*) (Supplementary Figure S5).

### 3.2 Experiments

For the multi-planar VA-Cohort and VA-ExtCohort, we report performance based on AC-PC predictions integrated from reformat-specific models (Sections 3.2.1–3.2.3). We note that this integration yielded only a modest improvement over axial reformat-specific predictions (detailed reformat-specific performance comparisons are provided in Supplementary Table S4 and Supplementary Figure S6). However, we utilize this integrated framework based on the rationale in Section 2.1 and alignment with the multi-planar nature of the AC-PC standardized ventriculomegaly features for the downstream NPH classification task (Section 3.3). For the axial-only UCSB-ExtCohort, we report performance based on the axial-reformat-specific predictions (Sections 3.2.1–3.2.3).

#### 3.2.1 Registration-guided 3D-UNet framework localizes with MRE < 2 mm on VA-cohort, outperforming benchmark methods

We employed a 5-fold cross-validation strategy with consistent patient-level train-test splits (80–20%) to develop and test both the registration-guided and baseline 3D-UNet frameworks on the VA-Cohort. For each reformat-specific registration-guided 3D-UNet, hyperparameters ( γ , α , *σ*, and initial learning rate [lr]) were tuned using nested cross-validation on secondary splits of the training sets (Supplementary Table S5). All models were trained with a batch size of 16 and for 25 epochs. As seen in the learning curves for the optimally selected hyperparameter configuration for each reformat-specific model (Supplementary Figure S7), this duration ensured training convergence; validation loss began to plateau around epoch 8 and remained stable through epoch 25, with no signs of overfitting. Random 3D rotations were subsequently applied to the final training (10 rotations per training set scan). We then integrated the AC-PC predictions from the optimized reformat-specific models to yield the framework’s final AC-PC coordinates (hereafter, integrated predictions).

**Figure 3:**
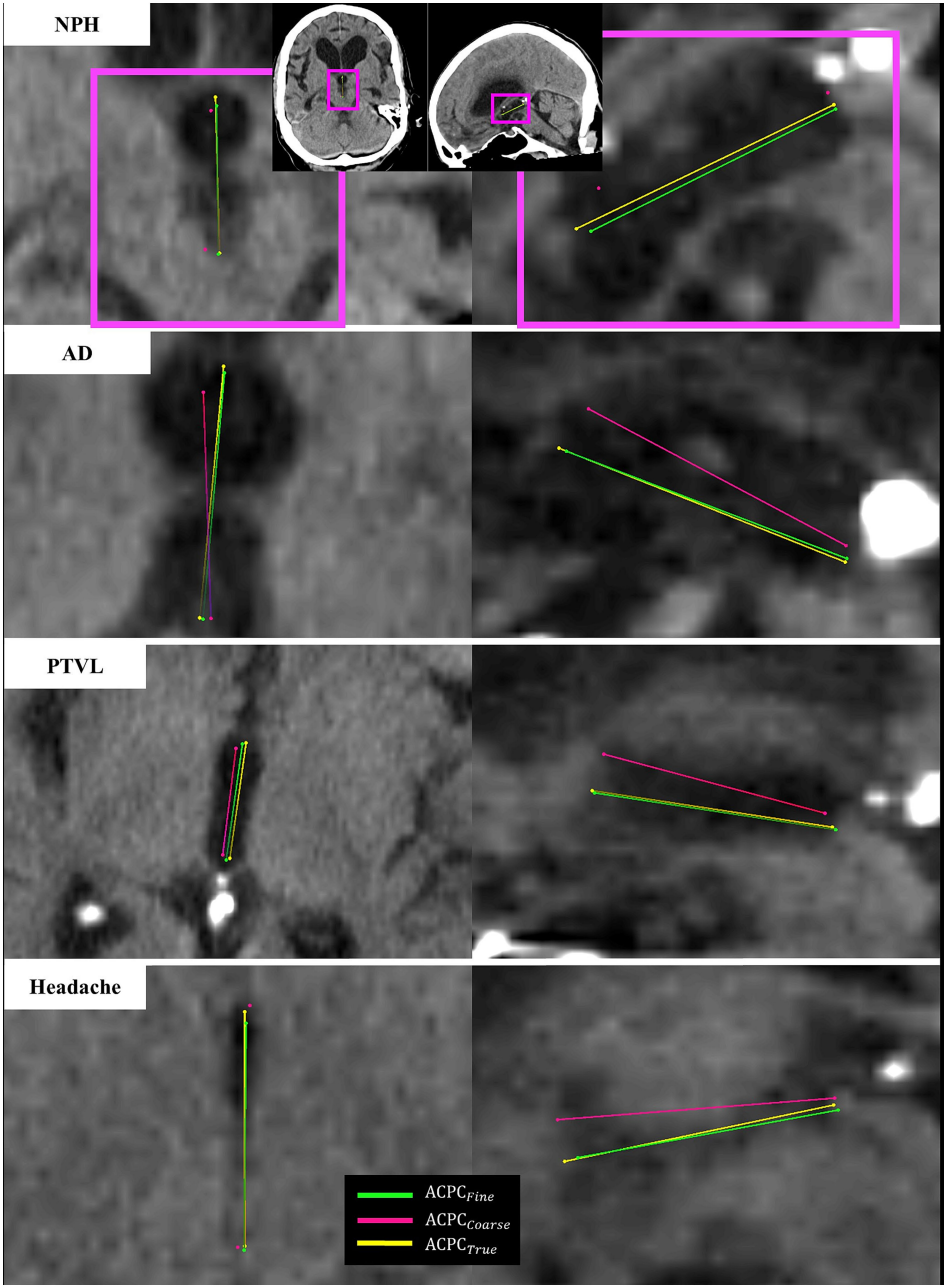
Cases of good AC-PC localization on representative high head-tilt test-set scans from the NPH, AD, headache, and PTVL groups of the VA-Cohort. Reference standard (*yellow*), coarse (*pink*), and fine (*green*) AC-PC localization are depicted for each neurological condition on cropped images from axial and sagittal reformats of each scan (as indicated by the *magenta* box for the NPH scan), are shown for each neurological condition. A line-segment connecting the AC-PC landmark predictions not being visible indicates that the specific line was out of the plane being shown. AC, Anterior Commissure, PC, Posterior Commissure, NPH, Normal Pressure Hydrocephalus, AD, Alzheimer’s disease; PTVL, Post-traumatic volume loss. Coarse/fine localized AC and PC REs were 4/2.33 and 1.27/0.44 mm, 4.63/1.78 and 0.69/0.46 mm, 4.38/2.17 and 0.84/0.88 mm, and 3.65/0.91 and 1.18/0.58 mm for the NPH, AD, PTVL, and headache scans, respectively. θ_yz_/θ_xy_ were 25.68°/1.51°, 21.65°/5.44°, 8.65°/8°, and 11.84°/0° for the NPH, AD, PTVL, and headache scans, respectively.

**Figure 4:**
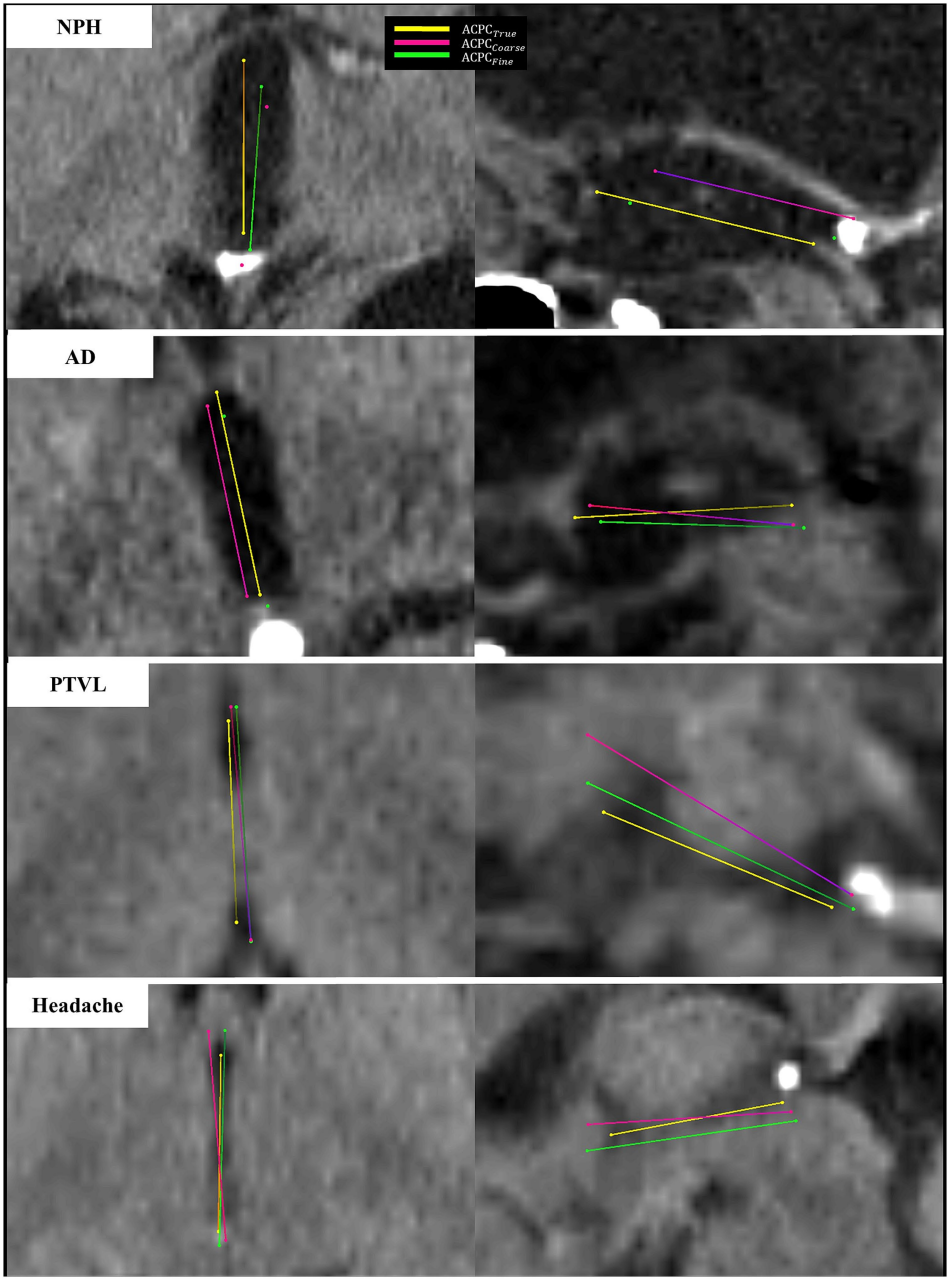
Cases of poor AC-PC localization on representative scans from the NPH, AD, headache, and PTVL groups of the VA-Cohort. Reference standard (*yellow*), coarse (*pink*), and fine (*green*) AC-PC localization are depicted for each neurological condition. Cropped images from the axial and sagittal reformats of each scan are shown for better visualization. A line-segment connecting the AC-PC landmark predictions not being visible indicates that the specific line was out of the plane being shown. AC, Anterior Commissure; PC, Posterior Commissure; NPH, Normal Pressure Hydrocephalus; AD, Alzheimer’s disease; and PTVL, Post-traumatic volume loss. Coarse/fine localized AC and PC REs were 9.68/6.75 and 5.85/3.31 mm, 2.49/2.76 and 3.12/3.08 mm, 6.7/2.41 and 2.91/2.28 mm, and 3.45/1.78 and 3.59/2.8 mm for the NPH, AD, PTVL, and headache scans, respectively. θ_yz_/θ_xy_ were 13.56°/0.11°, 3.26°/12.07°, 22.6°/2.35°, and 10.7°/0.8°, for the NPH, AD, PTVL, and headache scans, respectively.

These integrated AC-PC predictions resulted in test-set MREs of 1.64/1.49 mm. The 75th percentile and maximum AC-PC radial errors were 2/1.87 and 5.85/4.07 mm, respectively (Table 2). The 95% confidence intervals, estimated using bootstrapping, for the AC and PC radial errors were 1.57–1.73 and 1.42–1.56 mm, respectively. Note that the upper bounds of these 95% CIs were well below 3.2 mm, and in 95% of scans, the AC-PC radial errors relative to their reference-standard locations also fell within this 3.2 mm margin. This represents a clinically robust threshold: it falls well within the VA-Cohort’s native slice resolution and constitutes the upper limit before the distinctive power of downstream ventriculomegaly features begins to degrade (demonstrated in Section 3.3). While fine-stage AC and PC radial errors were moderately correlated with their coarse-stage counterparts (Pearson correlation [*r*] = 0.42 and 0.34, respectively), significant overall improvements were observed from the coarse to the fine localization stages (Table 2). The registration-guided 3D-UNet framework successfully localized the AC and PC across neurological conditions despite high head tilts (Figure 3), though poor localization still occurred in some edge cases (Figure 4).

**Table 2:** Performance comparison of proposed REGISTRATION-Guided 3D-UNet framework (fine-localization) vs. coarse-localization, and baseline-localization methods.

RE statistics	VA-Cohort AC	VA-Cohort PC	VA-ExtCohort AC	VA-ExtCohort PC	UCSB-ExtCohort AC	UCSB-ExtCohort PC
*Mean ± SD (mm)*						
Baseline	9.46 ± 4.2	9.95 ± 2.68	13.56 ± 5.64	15.52 ± 3.99	7.71 ± 3.32	10.66 ± 3.64
Coarse	3.3 ± 1.51	2.12 ± 1.03	3.9 ± 1.99	2.97 ± 1.6	2.61 ± 1.22	2.16 ± 1.01
Fine	1.64 ± 0.83	1.49 ± 0.72	2.42 + 1.06	1.79 + 0.86	2.31 + 0.98	1.93 + 0.78
*75th percentile (mm)*						
Baseline	12.26	12.04	14.9	18.07	10.13	13.55
Coarse	4.11	2.64	5.08	4.08	3.33	3
Fine	2.00	1.87	2.85	1.99	2.83	2.41
*Maximum (mm)*						
Baseline	23.44	18.59	33.67	26.23	14.88	19.47
Coarse	9.68	6.75	10.04	8.8	5.9	4.21
Fine	5.85	4.07	4.76	4.42	4.96	4.22
*RE_Coarse_–RE_Fine_*						
*d* (mm)	1.65	0.48	1.47	1.18	0.31	0.22
*q*	<0.001	<0.001	<0.001	<0.001	0.26	0.15
95% CI	[1.52, 1.78]	[0.54, 0.73]	[0.92, 2.03]	[0.81, 1.55]	[−0.24, 0.85]	[−0.06, 0.51]
*SDR_t_*						
t: 3.2 mm	94.9%	97.0%	82.5%	92.5%	83.7%	93.0%
t: 4.8 mm	99.1%	100.0%	100.0%	100.0%	97.7%	100.0%
t: 6.4 mm	100.0%	100.0%	100.0%	100.0%	100.0%	100.0%

For the multi-planar VA-Cohort and VA-ExtCohort, radial error (RE) statistics of the integrated reformat-specific AC-PC predictions from the three modeling approaches are reported. Predictions from using the axial reformats are reported for the axial-only UCSB-ExtCohort. For the baseline and registration-guided 3D-UNets, RE statistics are reported on predictions accumulated over the five held-out test-folds of the VA-Cohort (*n* = 427) and on inferred predictions on the VA-ExtCohort (*n* = 40) and UCSB-ExtCohort (*n* = 43). The average per-scan improvement in RE (d) from the coarse- to fine-localization methods was evaluated via paired *t*-tests. The Benjamini–Hochberg FDR-adjusted *p*-values (*q*) and unadjusted 95% confidence intervals (CI) are also presented. Note: Although integrated coarse results are provided to benchmark against integrated baseline and fine predictions, the fine-localization stage utilized reformat-specific coarse AC-PC locations to guide 3D patch selection for the 3D-UNets.

SDR_t_, Successful Detection Rate (percentage of scans with RE < t mm); SD, Standard Deviation; AC, Anterior Commissure; PC, Posterior Commissure; VA, Veterans Affairs; UCSB, University of California, Santa Barbara.

For each reformat-specific baseline 3D-UNet, six random hyperparameter sets were evaluated under 5-fold cross-validation. Due to the computational cost of whole-scan training, the best-performing hyperparameter set across the test folds was selected as the optimal set, bypassing the nested cross-validation approach (Supplementary Table S5). Although this test-set evaluation introduces an optimistic bias for the baseline, demonstrating that our strictly evaluated framework still outperforms this advantaged model underscores our method’s robustness. Mirroring the proposed framework, the primary baseline VA-Cohort results reflect integrated predictions from these optimal reformat-specific models. Baseline models were trained for 25 epochs with a batch size of 1 and no augmentations due to computational resource constraints. Test-set AC-PC MREs on integrated predictions from baseline localization were 9.46/9.95 mm (Table 2).

Because fatbACPC is restricted to axial scans, a direct comparison to our integrated AC-PC predictions was not possible. However, given that our integrated framework performs comparably to the axial-only results (Supplementary Table S4), the following axial reformat-specific observation suggests similar comparative trends hold for integrated predictions. The axial reformat-based AC-PC MREs for fatbACPC localization were 3.93/5.09 mm in the VA-Cohort, yielding higher localization errors than our coarse localization step on the same reformats (3.92/2.37 mm) (Table 3). This performance gap is expected, as fatbACPC relies on a linear approximation. In contrast, our coarse stage utilizes non-linear registration on skull-stripped brains, which better handles deeper intracranial anatomical mappings. Additionally, fatbACPC exhibited higher maximum errors. Consistent with our comparison of the coarse localization approach, the axial reformat-based fine 3D-UNet localization errors were notably lower than those of fatbACPC, reinforcing the necessity and robustness of the CNN-based refinement stage (Table 3).

**Table 3:** Performance comparison of proposed registration-guided 3D-UNet framework (coarse-to-fine localization) vs. fatbACPC for Axial Reformats.

RE statistics	VA-Cohort AC	VA-Cohort PC	VA-ExtCohort AC	VA-ExtCohort PC	UCSB-ExtCohort AC	UCSB-ExtCohort PC
*Mean ± Std.(mm)*						
fatbACPC	3.93 ± 4.44	5.09 ± 4.15	3.87 ± 1.97	4.32 ± 2.23	4.2 ± 5.66	4.81 ± 1.83
Coarse*	3.92 ± 1.69	2.37 ± 1.29	4.39 ± 2.27	3.2 ± 2.0	2.61 ± 1.22	2.16 ± 1.01
Fine*	1.79 ± 0.95	1.58 ± 0.75	2.41 ± 0.97	1.7 ± 1.04	2.31 + 0.98	1.93 + 0.78
*75th prcntl. (mm)*						
fatbACPC	4.35	5.74	4.8	5.24	5.07	5.74
Coarse*	4.88	3.02	5.05	3.95	3.33	3
Fine*	2.17	1.99	2.79	2.07	2.83	2.41
*Maximum (mm)*						
fatbACPC	40.87	43.27	9.37	10.67	38	9.6
Coarse*	10.3	9.37	11.38	11.3	5.9	4.21
Fine*	6.98	4.89	5.43	5.42	4.96	4.22

Radial error (RE) statistics for anterior commissure (AC) and posterior commissure (PC) localization are reported exclusively for axial reformats across all cohorts to ensure a direct comparison with the fatbACPC tool, which accepts only axial inputs.

*Indicates components of the proposed pipeline. Please note that three scans (two in the internal VA-Cohort [out of 427] and one in the UCSB-ExtCohort [out of 43]) were excluded from this comparison. We did not retain the original Digital Imaging and Communications in Medicine (DICOM) files required by fatbACPC for these scans. Attempts to bypass this limitation by providing files in the Neuroimaging Informatics Technology Initiative (NIfTI) format resulted in invalid (negative) voxel coordinates, indicating an alignment failure within the tool. VA, Veterans Affairs, UCSB, University of California, Santa Barbara.

#### 3.2.2 Registration-guided 3D-UNet framework provides MRE < 2.5 mm on external validation cohorts, outperforming benchmark methods

We validated the registration-guided 3D-UNet framework (trained on the VA-Cohort) on the VA-ExtCohort and UCSB-ExtCohort. Coarse AC-PC locations derived from the initial registration step were used to select the input patches for fine localization. Reformat-specific predictions were integrated for the VA-ExtCohort, whereas only the axial predictions were retained for the UCSB-ExtCohort. External performance showed an expected, marginal decrease relative to internal test-set results (Table 2). AC-PC MREs of the integrated predictions in the VA-ExtCohort were 2.42/1.79 mm. The 75th percentile and maximum AC-PC radial errors were 2.85/1.99 and 4.76/4.42 mm, respectively. The 95% confidence intervals (bootstrapping estimates) for the AC and PC radial errors in this cohort were 2.12–2.78 and 1.56–2.11 mm, respectively. In the axial-only UCSB-ExtCohort, AC-PC MREs were 2.31/1.93 mm, and 75th percentile and maximum AC-PC radial errors were 2.83/2.41 and 4.96/4.22 mm, respectively. The 95% confidence intervals (bootstrapping estimates) for the AC and PC MREs in this cohort were 2.04–2.66 and 1.71–2.19 mm, respectively. Not only were the upper bounds of these 95% confidence intervals well under 3.2 mm, but the predicted AC and PC locations also fell within 3.2 mm of the reference standard in >83% of scans across both cohorts.

Consistent with the VA-Cohort, fine AC and PC radial errors correlated with their coarse counterparts in the VA-ExtCohort (*r* = 0.49 and 0.70, respectively). In the UCSB-ExtCohort, only PC radial errors moderately correlated (*r* = 0.49). While significant coarse-to-fine AC-PC localization improvements were noted in the VA-ExtCohort, they were not pronounced in the UCSB-ExtCohort, which also had lower coarse radial errors than the VA-ExtCohort (and VA-Cohort; see Table 2). This is further examined in Section 3.2.3. Baseline models had test-set AC-PC MREs of 13.56/15.52 and 7.71/10.66 mm on the VA-ExtCohort and UCSB-ExtCohort, respectively (Table 2). The axial reformat-based AC-PC MREs for fatbACPC localization were 3.87/4.32 mm in the VA-ExtCohort and 4.20/4.81 mm in the UCSB-ExtCohort. Consistent with the trends and limitations observed in the VA-Cohort, these errors were higher than or comparable to our axial-reformat-based coarse localization performance and markedly higher than those of the proposed 3D-UNet refinement stage (Table 3).

To assess clinical feasibility, we recorded inference times for the fully automated registration-guided workflow on the VA-ExtCohort, which consisted of (1) preprocessing and image registration within 3D Slicer, and (2) 3D-UNet inference using an NVIDIA GeForce RTX 3080 GPU (10 GB VRAM). Image preprocessing and coarse-localization for the VA-ExtCohort (all three reformats) required 122.46 s/scan. Axial, coronal, and sagittal reformat-specific 3D-UNet inference times were 0.31, 0.36, and 0.31 s/scan, respectively. Total processing time was therefore ≤ 122.82 ss, which would reduce to ≤ 41.18 s/scan if reformats are processed in parallel.

#### 3.2.3 Consistent AC-PC localization across diverse scan parameters and clinical profiles

Across all datasets, the associations between AC-PC prediction radial errors and age, original CT slice thickness (before resampling to 1 mm^3^), and absolute head tilts (θ_yz_ and θ_xy_) were evaluated using Pearson correlation (r) (Table 4). Radial errors were uncorrelated with native slice thickness in the VA-Cohort and the VA-ExtCohort. Although there was a weak correlation between PC radial errors and slice thickness in the UCSB-ExtCohort (*r* = 0.31), this was not statistically significant. Head tilts in the (θ_yz_) and XY (θ_xy_) planes were also uncorrelated with radial errors except for a weak correlation between AC radial errors and θ_yz_ in the UCSB-ExtCohort (*r* = 0.36), which did not reach statistical significance, and a weak but significant association between PC radial errors and θ_xy_ in the VA-Cohort (*r* = 0.14). Age was uncorrelated with radial errors, except for a weak but significant association with PC radial errors in the VA-Cohort (*r* = −0.16).

**Table 4:** Factors associated with AC-PC localization by the registration-guided 3D-UNets.

Cohort: landmark	Native CT slice thickness	Head-tilt in YZ plane: θ_yz_	Head-tilt in XY plane: θ_xy_	Age
VA-Cohort				
AC	−0.05 [−0.14, 0.05]	0.02 [−0.08, 0.11]	0.01 [−0.08, 0.11]	−0.12 [−0.21, −0.02]
PC	−0.06 [−0.16, 0.03]	0.07 [−0.03, 0.16]	0.14 [0.05, 0.24]*	−0.16 [−0.25, −0.07]**
VA-ExtCohort				
AC	0.21 [−0.11, 0.49]	−0.17 [−0.46, 0.15]	−0.16 [−0.45, 0.16]	−0.15 [−0.44, 0.17]
PC	−0.1 4[−0.44, 0.18]	0.16 [−0.16, 0.45]	−0.19 [−0.47, 0.13]	0.12 [−0.2, 0.42]
UCSB-ExtCohort				
AC	0.17 [−0.14, 0.45]	0.36 [0.06, 0.59]	−0.1 [−0.39, 0.21]	0.16 [−0.15, 0.44]
PC	0.31 [0.01, 0.56]	0.1 [−0.21, 0.39]	0.12 [−0.19, 0.41]	−0.15 [−0.43, 0.16]

Pearson correlation coefficients (*r*) and their associated 95% confidence intervals (CI) between AC-PC radial errors (REs) and age, native slice-thickness, head tilt, and coarse-stage REs are reported.

Asterisks indicate Benjamini–Hochberg FDR-adjusted significance levels (*< 0.05, **< 0.01). Note that the average slice thickness from the axial, coronal, and sagittal reformats was used per scan to assess the correlation between slice thickness and REs for the VA-Cohort and the VA-ExtCohort. AC, Anterior Commissure, PC, Posterior Commissure.

To evaluate potential sex-based biases despite low female representation, we pooled subjects from the VA-Cohort and VA-ExtCohort (excluding the UCSB-ExtCohort due to the unavailability of sex information). A Welch’s two-sample *t*-test was used to compare fine AC-PC localization errors between male (*n* = 356) and female (*n* = 24) subjects. AC MREs showed no significant difference (*p* = 0.62; females vs. males: 1.81 vs. 1.71 mm). PC MREs were not significantly different but trended marginally higher for females (*p* = 0.087; females vs. males: 1.79 vs. 1.49 mm). This slight variance is likely confounded by age rather than sex; females in the VA cohorts were, on average a decade younger than males (females vs. males: 64.54 vs. 75.46 years), and PC radial errors exhibited a weak negative correlation with age in the VA-Cohort (*r* = −0.16).

**Figure 5:**
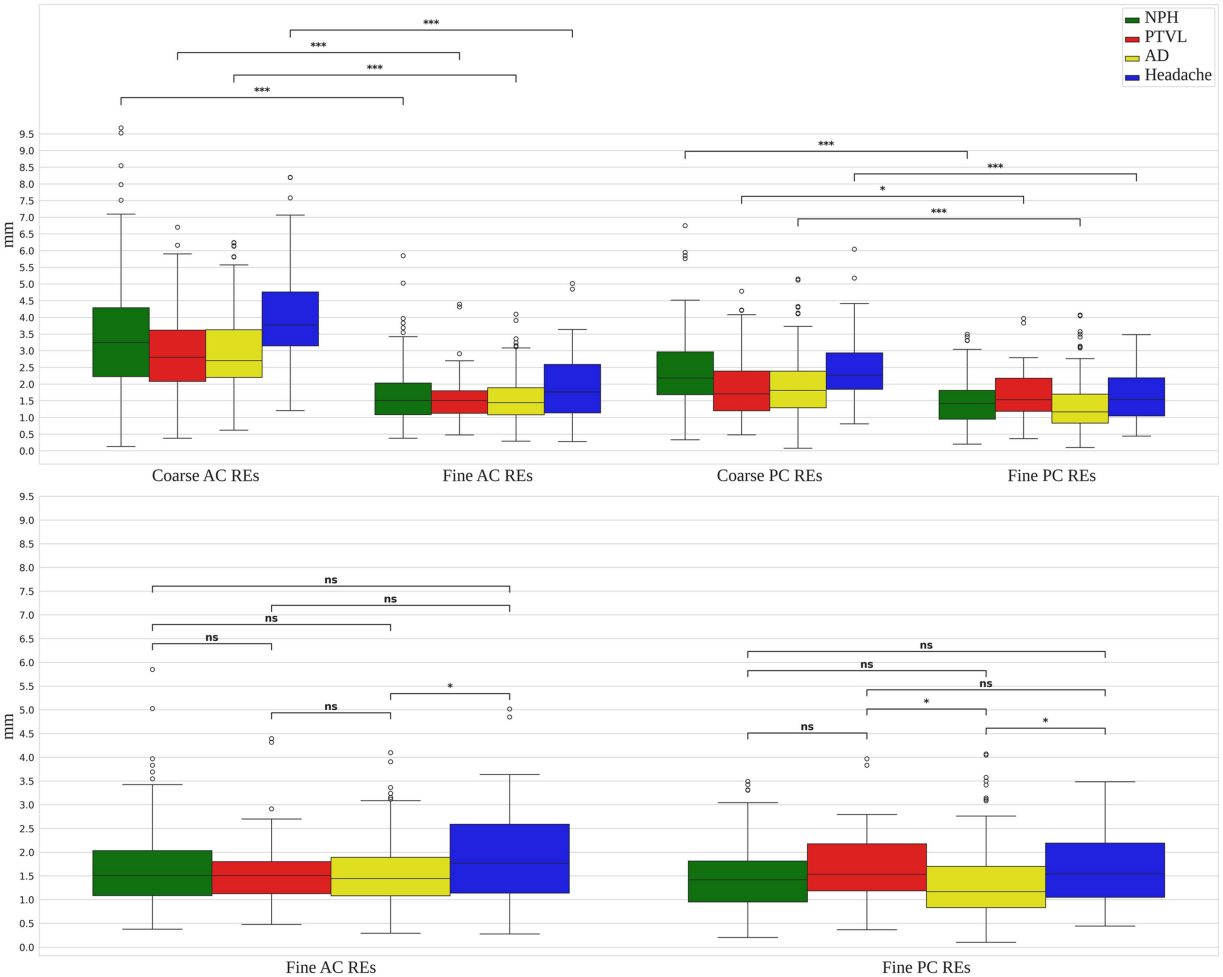
AC-PC localization by the registration-guided 3D-UNet framework across neurological conditions. Panel A depicts test-set AC-PC coarse-to-fine localization improvements across the neurological conditions in the VA-Cohort, evaluated using paired *t*-tests. Panel B depicts AC-PC fine localization performance across the neurological subgroups, compared via two-sample *t*-tests. Asterisks indicate statistical significance at the Benjamini–Hochberg corrected significance level (*q*). *q < 0.05, ***q < 0.001, ns: not significant. RE, Radial Error, NPH, Normal Pressure Hydrocephalus, AD, Alzheimer’s disease, PTVL, Post-traumatic volume loss, AC, Anterior Commissure, PC, Posterior Commissure.

Paired *t*-tests to compare the average scan-wise reduction in radial error from the coarse to fine localization stages revealed significant improvements across all neurological conditions in the VA-Cohort (Figure 5). Two-sample *t*-tests revealed that fine-stage AC-PC localization performance was similar across neurological conditions, except for significant differences (d) in AC MREs between Alzheimer’s disease and headache (*d*: −0.36 mm, *p* = 0.01, 95% CI: [−0.57, −0.14]), and PC MREs between Alzheimer’s disease and headache (*d*: −0.3 mm, p = 0.01, 95% CI: [−0.49, −0.1]), and Alzheimer’s disease and post-traumatic volume loss (*d*: −0.32 mm, p = 0.01, 95% CI: [−0.53, −0.12]).

The UCSB-ExtCohort’s NPH subgroup showed a trend toward improvement from the coarse to fine localization stages (*n* = 13; AC-PC MREs dropped from 3.47/2.02 to 2.42/1.58 mm; *p* = 0.05/0.10), likely missing statistical significance due to the small sample size. Conversely, its normal control (NC) subgroup showed no improvement (*n* = 30; from 2.24/2.22 to 2.26/2.09 mm; *p* = 0.97/0.47). We hypothesize this lack of improvement in the NC subgroup is due to a ‘ceiling effect’ driven by the advanced age of these normative aging adults. In the VA-Cohort, older age was weakly correlated with better coarse localization (AC-PC *r* = −0.26/−0.17; *p* < 0.001). Additionally, the VA-Cohort’s headache patients (normal proxy), who were significantly younger than the UCSB-ExtCohort’s NC patients (median age: 58 vs. 70 years; *U* = 1910, *p* < 0.001), demonstrated significant improvement in coarse-to-fine localization (Figure 5). Therefore, coarse registration in the UCSB-ExtCohort’s NC patients was likely already near-optimal, precluding further refinement during the fine stage.

### 3.3 Proposed framework eliminates the manual AC-PC localization bottleneck for Ventriculomegaly feature computation to classify NPH

Within the VA-Cohort-CS, our framework achieved AC-PC MREs of 1.67/1.50 mm. These errors were comparable to those of the remaining VA-Cohort patients (1.60/1.45 mm; *p* = 0.46/0.55), confirming the absence of inadvertent selection bias. To isolate the impact of AC-PC localization on NPH classification performance, the midline points required for radiological feature computation were manually defined across all experiments.

**Figure 6:**
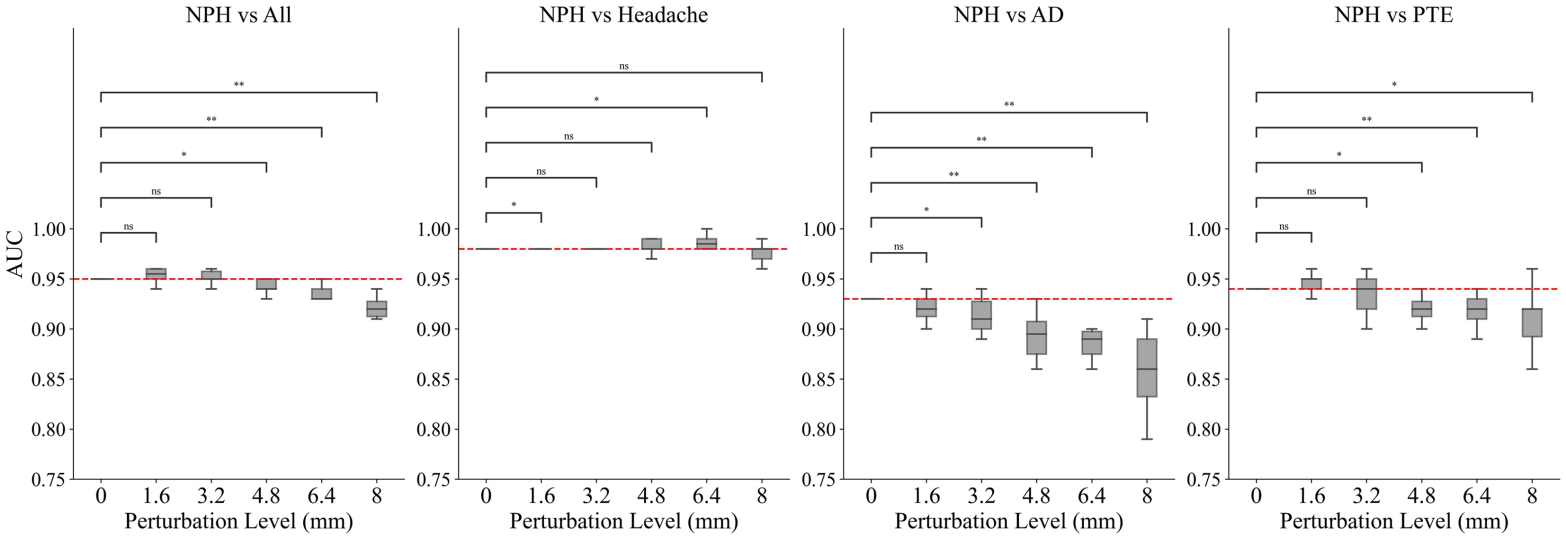
Impact of accurate AC-PC localization on NPH classification in the VA-Cohort-CS. Test-set AUC changes in the classification of NPH from headache/AD/PTVL (‘All’ refers to the NPH-vs-rest multi-class classification scenario) based on ventriculomegaly features computed using the reference-standard AC-PC (Perturbation Level = 0 mm) vs. randomly perturbed AC-PC (boxplots of 10 repeats at each noise/perturbation level). Asterisks indicate statistical significance at the Benjamini–Hochberg corrected significance level (*q*). *q < 0.05, **q < 0.01, ns, not significant. NPH, Normal Pressure Hydrocephalus; AD, Alzheimer’s disease; PTVL, Post-traumatic volume loss; AC, Anterior Commissure; PC, Posterior Commissure.

In the perturbation study, artificially introduced AC-PC errors significantly degraded test-set NPH classification AUCs at MREs ≥ 3.2 mm (vs. Alzheimer’s disease), ≥ 4.8 mm (vs. post-traumatic volume loss), and ≥ 6.4 mm (vs. headache patients) (Figure 6). Notably, our framework’s radial errors for 95% of the VA-Cohort scans were well within this critical 3.2-mm threshold, beyond which the combined distinctive capability of the AC-PC-referenced ventriculomegaly features began to degrade. Features-PredACPC (computed using our framework’s predictions) yielded an AUC of 0.95, an accuracy of 89%, a sensitivity of 92%, and a specificity of 87% for multi-class NPH classification (Table 5). McNemar’s tests confirmed this performance showed no significant difference from that of models using the manual Features-RefACPC (Table 5) across both multi-class (*p* = 0.22) and binary settings (*p* = 0.69, *p* > 0.99, and *p* > 0.99 for post-traumatic volume loss, Alzheimer’s disease, and headache, respectively).

**Table 5:** NPH classification using ventriculomegaly features standardized with AC-PC predictions from the registration-guided 3D-UNet framework.

Classification	AUC	Accuracy	Precision	Sensitivity	Specificity	*p*-val
	Pred.| Ref.	Pred.| Ref.	Pred.| Ref.	Pred.| Ref.	Pred.| Ref.	
NPH vs. AD	0.92 | 0.93	85% | 86%	87% | 87%	83% | 84%	87% | 87%	>0.99
NPH vs. PTVL	0.94 | 0.94	91% | 89%	90% | 89%	94% | 91%	87% | 87%	0.69
NPH vs. headache	0.98 | 0.98	99% | 99%	98% | 98%	100% | 100%	98% | 98%	>0.99
NPH vs. All	0.95 | 0.95	89% | 87%	73% | 70%	92% | 91%	87% | 86%	0.22

Performance measures for classification between NPH (*n* = 64), AD (*n* = 62), PTVL (*n* = 53), and headache patients (*n* = 59), using ventriculomegaly features derived in reference to AC-PC predictions from our methodology (Pred.) compared to using ground-truth AC-PC annotations (Ref.) on the VA-Cohort-CS (*n* = 238). Measures are reported by accumulating predictions over held-out test sets from 5-fold cross-validation. *p*-values from McNemar’s tests (p-val) compare class distributions of predicted neurological condition labels in both scenarios are shown.

NPH, Normal Pressure Hydrocephalus; AD, Alzheimer’s disease; PTVL, Post-traumatic volume loss; AUC, Area Under the Receiver Operating Characteristic Curve.

Paired *t*-tests confirmed that individual feature values did not differ significantly between Features-PredACPC and Features-RefACPC, except for negligible differences in Evans Index-y and Evans Index-z (*d* ≈ −0.003; Supplementary Table S7). Furthermore, univariate analysis verified that classification was not driven by circularity bias from the Evans Index-x > 0.3 diagnostic inclusion criterion, as Evans Index-z and maximum eccentricity of the lateral ventricles outperformed Evans Index-x in distinguishing NPH from Alzheimer’s disease/post-traumatic volume loss (Supplementary Table S8). Finally, the standalone predictive power of the AC-PC independent cerebrospinal fluid to brain volume ratio feature (AUCs of 0.78 and 0.90, for NPH vs. Alzheimer’s disease and post-traumatic volume loss, respectively) was substantially lower than the combined Features-PredACPC (AUCs of 0.92 and 0.94), reinforcing the necessity of accurate AC-PC localization for accurate clinical screening of NPH.

## 4 Discussion

Our registration-guided 3D-UNet framework achieved AC-PC MREs of 1.64/1.49 mm on 1-mm^3^-resampled internal test-set scans, and the predicted AC and PC were each within 3.2 mm of the reference standard in > 95% of scans. Although the AC-PC radial errors in the 1-mm^3^ resampled external validation cohorts (2.42/1.79 and 2.31/1.93 mm) were slightly higher as expected due to domain shift, the landmark predictions were each within 3.2 mm of the reference standard in > 83% of scans. This 3.2-mm benchmark is significant for two primary reasons. First, this threshold is smaller than the native axial slice thickness of the 5-mm cohorts (VA-Cohort and UCSB-ExtCohort) and approximately equal to that of the 3-mm cohort (VA-ExtCohort), thereby demonstrating that the framework’s localization accuracy aligns with the through-plane resolution of routine CT scans. Given that the compact AC and PC structures measure approximately 2–3 mm along their short axes at the midsagittal plane (Ardekani and Bachman, 2009; Choi et al., 2013), these landmark predictions are subsumed within a single slice of routine CT, and localization within 3.2 mm indicates the model has localized the targets within tight bounds. Second, 3.2 mm represents a critical clinical threshold; errors exceeding this limit induced a significant drop in the downstream NPH classification accuracy of AC-PC-referenced ventriculomegaly features.

Our framework demonstrates robustness across a wide range of clinical profiles, achieving consistent fine-stage localization performance across varied neurodegenerative conditions. Notably, while coarse localization provides sufficient accuracy for older, normal-subject scans, where age-related atrophy naturally enhances structural definition and contrast (UCSB-ExtCohort’s NC), the fine-localization component serves as a critical refinement step for more challenging phenotypes. Specifically, it ensures high performance in younger, normal cases that lack this age-related structural contrast (VA-Cohort’s headache patients), as well as in cases presenting with pathological structural degeneration (VA-Cohort’s NPH, Alzheimer’s disease, post-traumatic volume loss; VA-ExtCohort; and UCSB-ExtCohort’s NPH). Importantly, the 3D-UNet refinement significantly reduced localization errors from the coarse stage across all neurological subgroups of the VA-Cohort. However, in cases of suboptimal localizations even in the fine-stage, visual inspection suggested that severe ventricular distention in NPH, structural distortion due to severe atrophy in Alzheimer’s disease, and lower-contrast anatomies of younger post-traumatic volume loss and headache patients without the structural definition induced by age-related atrophy were primary drivers of error (Figure 4). Furthermore, our framework’s performance was robust to both head tilts and patient age, neither of which notably affected radial errors. Native slice thickness similarly did not impact radial errors, ruling out scan resolution as a limiting factor.

AC-PC localization via our registration-guided 3D-UNet framework significantly outperformed inferred localization using fatbACPC, the only dedicated CT-based solution for AC-PC alignment (Rubbert et al., 2021). Because fatbACPC relies on a purely registration-based approach (a rigid-transformation approximation of an affine transform), it is inherently limited when mapping deep intracranial landmark predictions, such as the AC and PC, in scans with significant pathological variations. Notably, our coarse localization step alone achieved performance comparable to or slightly better than fatbACPC. This may be attributed to our use of non-rigid transformations on skull-stripped images. As supported by existing MRI-based literature, while skull stripping improves registration accuracy by allowing the algorithm to focus on the relevant neuroanatomy (Hoopes et al., 2022), non-linear registration can better handle severe structural distortions, such as hydrocephalus (Ragguett et al., 2024). Within our framework, while the correlation between coarse and fine-stage radial errors emphasized the need for well-chosen input patches to the 3D-UNets, the 3D-UNets significantly refined localization within these registration-guided windows. Additionally, although integrated predictions were slightly superior, axial reformats yielded performance that was highly comparable, demonstrating the framework’s broad applicability under axial-only constraints. Finally, the framework also significantly outperformed the baseline localization approach (the standalone global 3D-UNet without registration guidance), highlighting the utility and necessity of the proposed coarse-to-fine localization strategy.

Previous studies evaluating diagnostic radiological markers of NPH have relied on inefficient manual AC-PC localization (Kockum et al., 2020; Kadaba Sridhar et al., 2024b) or bias-inducing non-linear registration (Takahashi et al., 2017). Our framework eliminated the bottleneck of manual AC-PC localization, which is required to standardize diagnostic-grade ventriculomegaly features for the classification of NPH (Nakajima et al., 2021; Pyrgelis et al., 2023; Behndig et al., 2025), a surgically reversible dementia that is frequently misdiagnosed as its atrophic mimics. Prior research has demonstrated that accurate selection and rotation of the measurement plane, relying on AC-PC localization, affect the diagnostic utility of diagnostic utility of guideline-recommended features such as the Evans Index and Callosal Angle (Lee et al., 2021; Ryska et al., 2021). Localization errors exceeding 3.2 mm compromised the ability of AC-PC standardized features to distinguish between hydrocephalic and atrophic ventriculomegaly. By achieving an MRE below this threshold on more than 83% of scans across all cohorts, our proposed framework provides the robust feature standardization necessary for accurate NPH diagnosis. Features derived using our framework’s AC-PC predictions matched or exceeded (AUC = 0.95) complex neural networks (Zhang et al., 2022; Haber et al., 2023; Songsaeng et al., 2023) in detecting NPH on CT, while also offering interpretation for clinical utility. Including diverse pathological groups (Alzheimer’s disease and post-traumatic volume loss) ensured that the NPH classification task was not biased by easily differentiable severe hydrocephalic ventriculomegaly vs. normal cases only.

Study limitations include the predominantly male sample due to the demographic characteristics of the veteran population (Wilmoth et al., 2020) and the lack of sex information for the UCSB-ExtCohort. While comparisons between the male and a few female subjects in our cohorts showed no significant sex biases (with minor PC error variances likely confounded by the female group being, on average, a decade younger), the framework’s generalizability remains to be validated on a larger female cohort with a more representative age range, particularly including older patients. The test-only datasets contained hydrocephalus and normal scans only, which limited validation on scans with Alzheimer’s disease and post-traumatic volume loss. The small sample sizes of these test-only datasets (*n* = 40 and *n* = 43) limited the statistical power of our subgroup analyses to detect subtle but potentially meaningful effects, such as improvements from the coarse-to-fine localization stages, or weak correlations between localization errors and acquisition factors. Additionally, as our study’s scope was limited to chronic neurological conditions that require AC-PC standardized imaging markers, we excluded acute scans that are routinely evaluated visually. Extending this method to acute scans will require careful evaluation of the proposal patches generated by the registration step. Automatic checks for consistency with anatomical domain knowledge, such as the distance of the predicted AC and PC locations from the brain centroid the AC always being anterior to the PC, and the distance between the AC and PC locations, may be used for quality checks. Registration failures of >40° YZ head-tilt scans highlighted challenges in extreme patient positioning, which necessitate radiologist oversight and intervention for such outliers. Although the lack of correlation between native slice thickness and localization error likely reflects our design choice to generate reference standards from resampled 1 mm^3^ volumes (which homogenizes input resolution), the high inter-annotator agreement (<1 mm MRE) validated the consistency and reproducibility of these targets. However, the potential performance gains from native high-resolution acquisitions from modern scanners remain to be quantified.

Our AI-enabled framework localizes the AC and PC on 1-mm^3^-resampled routine CT (native slice thicknesses of 3 and 5 mm), achieving a clinically relevant localization limit of 3.2 mm in over 83% of scans. While high-resolution CT or MR offers ideal resolution for submillimeter accuracy, the accuracy achieved by our framework enables reliable computation of standardized ventriculomegaly markers to discriminate hydrocephalus from atrophy. With a prediction time under 42 s, this tool is well-suited for high-volume settings like emergency departments, where routine CT often serves as the initial evaluation for elderly patients presenting with balance issues, falls, or impaired mentation. While the automated preprocessing and skull-stripping pipeline was successful across all scans in our evaluation cohorts, real-world clinical integration should include clinician oversight for visual verification of the localized landmark predictions, final head alignment, and downstream feature computation. Once validated in larger, sex-balanced external cohorts, this tool can support clinical decision-making, potentially avoiding unnecessary MRIs by maximizing the utility of CT, which is quicker, more widely accessible, and has fewer contraindications. In its current form, our proposed methodology, combined with existing computational pipelines for ventriculomegaly features on CT, provides an NPH classification workflow that requires minimal manual intervention. Although mid-sagittal plane localization was beyond the study’s scope, integrating the current framework with an automatic midline detection algorithm in the future would fully automate clinical deployment and may also improve registration in scans with acute findings. Our framework may also be adapted to CT with implants and leverage improved scanner resolution for higher precision AC-PC localization.

## Data Availability

The datasets presented in this article are not readily available because the data analyzed in this study is subject to U.S. Department of Veterans Affairs (VA) regulations and cannot be shared publicly. Access to both raw and derived imaging data may be authorized to personnel operating within the VA system. Requests to access the datasets should be directed to uzma.samadani@va.gov.
