# Supplementary material for: Automatic coarse-to-fine AC-PC localization on CT using registration-guided 3D-UNets
Source: Front Neuroinform. 2026 Jul 21;20:1853412. doi: 10.3389/fninf.2026.1853412 (PMC13445414; doi:10.3389/fninf.2026.1853412)
Supplement: Supplementary file 1 [file Data_Sheet_1.PDF]

Supplementary Material

Table of Contents

1. **Table S1.** CT vs. MRI Trends (2014-2024) .... Page 2
2. **Table S2.** Inclusion-Exclusion Criteria for the VA Cohorts .... Page 2
3. **Supplemental Note 1.** CT Image Acquisition Details .... Page 3
4. **Supplemental Note 2.** Obtaining the AC-PC Reference Standard .... Page 3
   Figure S1. Multi-Annotator AC-PC Annotation Workflow
5. **Figure S2.** Illustration of Coarse localization via Image Registration .... Page 5
6. **Figure S3.** Depiction of AC-PC referenced computation of ventriculomegaly features. .... Page 6
7. **Supplemental Note 3.** Impact of Precise AC-PC Localization on NPH Classification .... Page 6
8. **Figure S4.** End-to-End Evaluation using 5-fold CV .... Page 7
9. **Table S3.** Inter-Annotator Agreement in the VA-Cohort .... Page 8
10. **Figure S5**. Multi-Annotator Agreement in the VA-Cohort .... Page 8
11. **Table S4.** Integrated vs. Reformat-Specific Coarse- and Baseline-Localization .... Page 9
12. **Figure S6.** Coordinate Errors in Integrated vs. Reformat-Specific Fine-Localization .. Page 10
13. **Table S5.** Selected Hyperparameters (HPs) .... Page 11
14. **Figure S7.** Training and validation loss trajectories .... Page 11
15. **Table S6.** Predicted/True Class Frequencies in NPH Classification .... Page 12
16. **Table S7.** Ventriculomegaly Features Using Predicted vs. Reference AC-PC .... Page 13
17. **Table S8.** Feature-specific NPH Classification .... Page 13

Table S1. **CT vs MRI Trends (2014–2024).** CT vs MRI trends for patients with suspected dementia and unlikely intracranial injury found in the Veterans Affairs Informatics and Computing Infrastructure (VINCI), between 2014 and 2024. Neurological conditions were inferred based on international classification of disease codes as opposed to rigorous chart-review, so these estimates should be considered as approximate. Even if the true number of patients who had a CT-only was reduced by 50% with chart-review confirmation of the neurological disease (~6.2k), the number of patients with a CT-only was 4x as that of those with MRI-only. FTD = Frontotemporal dementia, VaD = Vascular dementia, AD = Alzheimer's disease, DLB = Dementia with Lew Bodies, NPH = Normal Pressure Hydrocephalus.

Neurological Condition	CT-Only	MRI-Only	CT-and-MRI	CT-or-MRI	% CT-Only	% MRI-Only	% CT-and-MRI
FTD	2,992	462	594	4,048	73.9%	11.4%	14.7%
VaD	27,230	3,169	5,089	35,488	76.7%	8.9%	14.3%
AD	24,576	2,602	3,024	30,202	81.4%	8.6%	10.0%
DLB	5,875	807	1,033	7,715	76.2%	10.5%	13.4%
NPH	1,906	277	418	2,601	73.3%	10.6%	16.1%
**Average**	**12,516**	**1,463**	**2,032**	**16,011**	**78.2%**	**9.1%**	**12.7%**

Table S2. Inclusion-Exclusion Criteria for the VA Cohorts. ICD=International Classification of Disease, CPT=Current Procedural Terminology, NPH=Normal Pressure Hydrocephalus, AD=Alzheimer's disease, SDH=subdural hemorrhage, PTVL=Post-traumatic volume loss, PD=Parkinson's disease, FTD=Frontotemporal dementia, DLB=Dementia with Lewy bodies, VaD=Vascular dementia, PSP=Progressive supranuclear palsy, ICH=Intracranial hemorrhage, %=wildcard (pattern matching), and EI=Evans Index. *The VA-Cohort-CS, a subset of the VA-Cohort, was first subject to the VA-Cohort's inclusion-exclusion criteria and further qualified via chart-review.

Dataset	Inclusion Criteria	Exclusion Criteria	Scan Criteria
VA-Cohort	*Diagnostic Codes*	*Diagnostic Codes & Pattern Matching on Patient Charts*	
NPH	ICD 9/10: 331.5/G91.2	AD or SDH	Hydrocephalus, No shunt
AD	ICD 9/10: 331/G30.0, G30.1, G30.8, G30.9	NPH or SDH	Atrophy
PTVL	ICD 9/10: 852.2%, 852.3%/ S06.5X% (Traumatic SDH)	NPH or AD	Almost resolved/ no chronic SDH & volume loss
Headache	ICD 9/10: 784.0/R51.9	NPH, AD, or SDH	No hydrocephalus, atrophy, or intra-cranial lesions
Common criteria		PD, FTD, DLB, VaD, or intracranial tumor	

Dataset	Inclusion Criteria	Exclusion Criteria	Scan Criteria
VA-ExtCohort	*Procedural Codes and Chart-review*		
Hydro-cephalus	CPT: 220, 61070, 62200, 62256, 78645, 62180, 62160, 62190, 62192, 62223, 62230, 62258, T6089, 62201, 62220, 62252; Chart review: Shunted hydrocephalus		No acute findings (including significant midline shift), intracranial lesions, or shunt in scan.
VA-Cohort-CS*	Chart-review	*Chart-review*	
NPH	Impaired gait, and cognition or urination; 60+ years of age; ventriculomegaly in radiology charts	Secondary hydrocephalus, AD, or SDH excluding post-shunt cases in NPH	EI > 0.3
AD	Suspected AD	NPH or SDH	
PTVL	Chronic SDH due to trauma	NPH or AD	
Headache	Headache with no known neurological disorder	NPH, AD, or SDH	
Common criteria		PD, aqueductal stenosis, meningitis, encephalitis, PSP, intracranial tumor, FTD, DLB, ICH excluding SDH, VaD, stroke, or other neurological disorders explaining the symptoms	

Supplemental Note 1. CT Image Acquisition Details

VA-Cohort scans were primarily acquired on Philips (48%), Siemens (46%), Toshiba (6%) or Canon (<1%, 1 scan) scanners (median parameters: tube current 213 mAs, collimation 19.2 mm, peak potential 120 kVp, CT dosage index 56.6 mGy). VA-ExtCohort scans were generated from Toshiba (n = 35%), Siemens (15%), General Electric (10%), Philips (8%), or Canon (3%) scanners (29% unknown) with a median tube current of 255 mAs. Remaining VA-ExtCohort scanner parameters and all UCSB-ExtCohort scanner parameters were unavailable due to de-identification. For the VA-Cohort, median in-plane resolutions were 0.47, 0.43, and 0.44 mm for axial, coronal, and sagittal reformats, respectively. In the VA-ExtCohort, resolutions were 0.47, 0.43, and 0.43 mm for axial, coronal, and sagittal reformats, respectively. Resolution for the UCSB-ExtCohort's axial reformats was 0.49 mm.

Supplemental Note 2. AC-PC Reference Standard Annotation Protocol

Annotations were created on fully deidentified scans. Manual AC-PC annotations were created via 3D Slicer. We leveraged the availability of all orthogonal CT reformats (axial, coronal, and sagittal) in the VA-Cohort and VA-ExtCohort to generate the AC-PC reference standards. This approach improved visualization compared to individual reformats with their natively anisotropic resolutions. In contrast, the UCSB-ExtCohort was limited to axial reformats; therefore, we rendered sagittal and coronal views from the axial volume to enable the same annotation protocol.

Initial AC and PC estimates on the central intercommissural line (CIL), along with 20 midsagittal plane (MSP) points was marked on each scan using visual cues from all three orthogonal reformats (axial, sagittal, and coronal). As the commissures were generally more visually distinguishable post AC-PC and MSP alignment (AC-PC line horizontal and midline vertical), 3D Slicer's "ACPC Transform" module was used to align the scans (by creating a linear alignment transform, T_A) using the AC, PC, and MSP annotations in the native image spaces. Necessary adjustments were made to the AC and PC landmarks in aligned spaces while ensuring that the left-right (x) and inferior-superior (z) coordinate deviations between the AC and PC were minimal (< 1 mm; this was based on the rationale that in a perfectly AC-PC aligned scan, the x and z coordinates of the AC and PC will be the same), and reflected back to native spaces using T_A^{-1}, before recording them.

Figure S1. Multi-Annotator AC-PC Annotation Workflow. Process depicting the creation of the AC-PC reference standard on all datasets used in this study. PA = Primary Annotator, SA = Secondary Annotator, RE = Radial Error (Euclidean distance between PA and SA annotations). *RE > 1.7 mm was chosen to flag scans with absolute deviation higher than 1mm in x/y/z coordinates. #The smaller size of SA1's subset was due to time constraints and availability, rather than intentional study design.

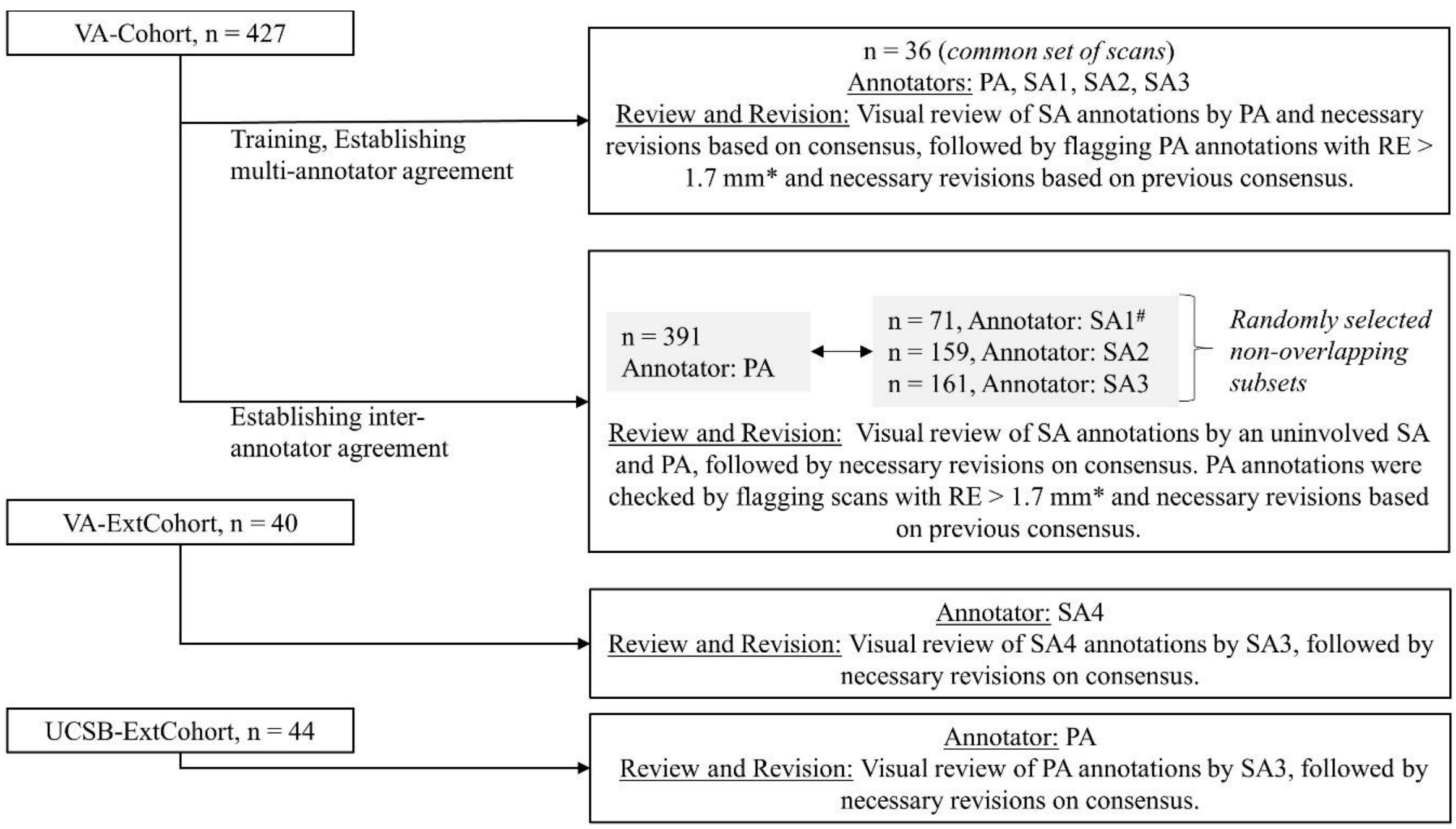

The workflow for creation of the AC-PC reference standard for the study datasets are in Figure S1. The reference standard was established through a multi-stage process. The Primary Annotator (PA) generated initial annotations for the VA-Cohort. To ensure adherence to protocol and assess inter-annotator agreement, independent secondary annotations were generated by trained secondary annotators (SAs) 1, 2, and 3 for the VA-Cohort. A common set of randomly sampled 36 scans from the VA-Cohort were initially annotated by the SAs for training on and familiarization with the software platform and annotation protocol. Multi-annotator agreement between the PA and SAs was also evaluated on this set of scans. Next, the SAs (blinded to PA annotations) annotated mutually exclusive, randomly sampled subsets of the VA-Cohort. These were visually audited for protocol adherence by an uninvolved SA in conjunction with the PA, with discrepancies resolved through joint review and mutual consensus. Finally, PA annotations which deviated from finalized SA annotations by more than 1 mm in x/y/z coordinates were flagged quantitatively, reviewed, and harmonized to protocol if

necessary, based on the finalized consensus standard. SA4 annotated the VA-ExtCohort and the PA annotated the UCSB-ExtCohort. SA3 conducted a systematic audit of these annotations. Any protocol deviations identified were recorded and harmonized via consensus between the reviewer (SA3) and annotator (SA4/PA).

Figure S2. Illustration of Coarse localization via Image Registration. Panel A shows the CT template with known AC-PC landmarks ($AC_{Template}$, $PC_{Template}$) on the central intercommissural line, and the input CT which requires AC-PC localization. Panel B shows the nonlinear transformation, T^{-1} (*yellow* contours), learned via image registration (morphs the template onto the input CT); the mapping of $AC_{Template}$- $PC_{Template}$ (*yellow*) onto the input CT as AC_{Coarse}-PC_{Coarse} (*pink*); and consequently, the selection of input patches (*white*) for the 3D-UNet which is trained to finely localize the AC-PC on the input scan (reference standard AC-PC are in *green*). Note that processing was in 3D although 2D slices are used for illustration.

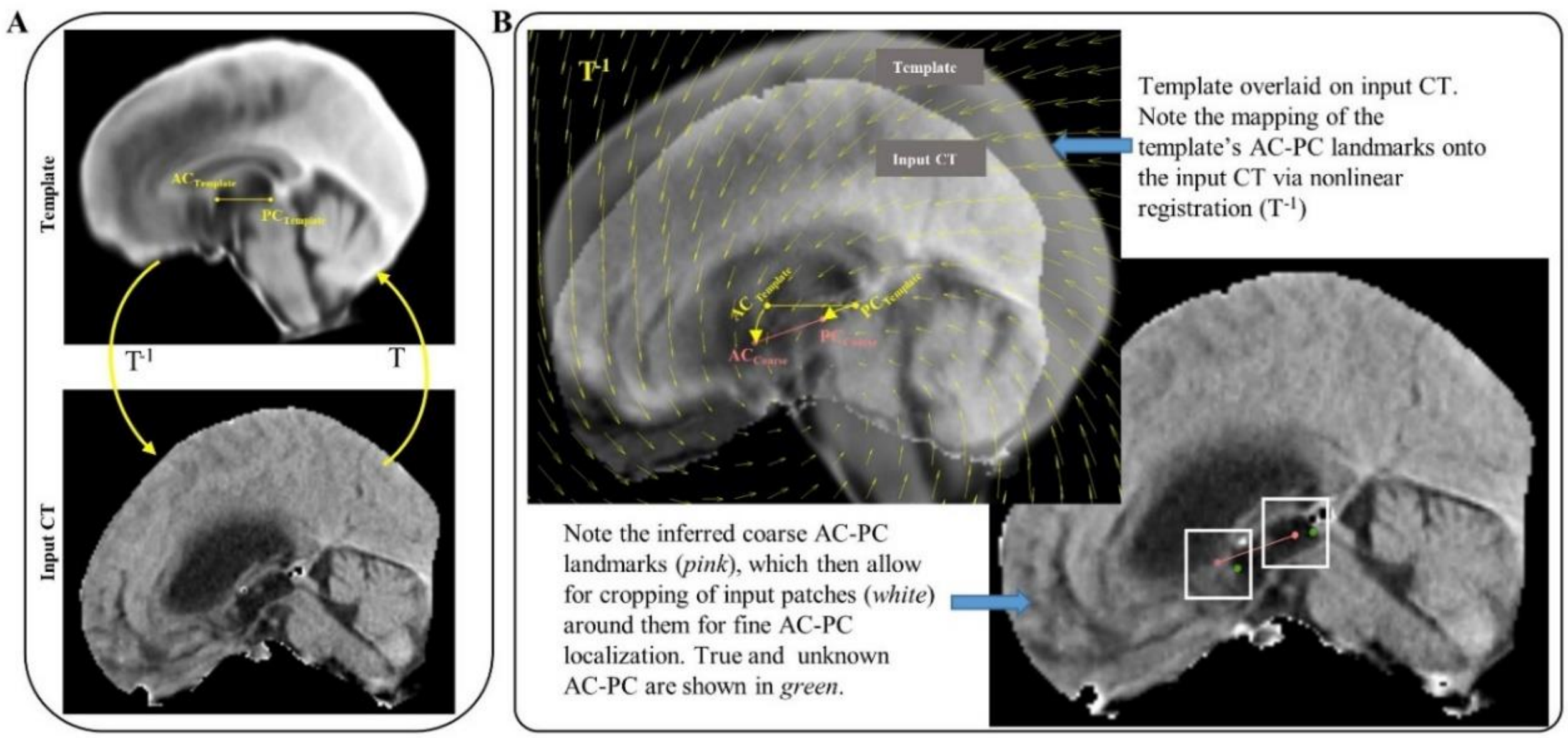

Figure S3. Depiction of AC-PC referenced computation of ventriculomegaly features. Each feature is depicted on the plane of measurement embedded in the 3D brain (*brown*) and ventricular space (*blue*), along with the reference AC-PC line (*green*). Top row (left to right): The Evans indices in the x and y directions (EI-x and EI-y), normalized maximum third ventricle width (NMax-3VW), and proxy Splenial Angle (p-SA) are assessed on axial slices parallel to the AC-PC plane. Bottom row (left to right): The Maximum Eccentricity of the Lateral Ventricles (MaxEccLV), Callosal Angle (CA), and the Evans index in the z direction (EI-z) are assessed on coronal slices perpendicular to the AC-PC plane. Additionally, each feature is assessed in reference to standard locations determined by the AC and PC's spatial coordinates: the EI-x, EI-y, and NMax-3VW as normalized measures of the maximum frontal horn (MFH) width (MFH-x/Max-x), MFH length (MFH-y/Max-y), and 3rd ventricle width (3V-Max-x/Max-3VLevel-BW) on a range of axial slices defined by the AC-level; the MaxEccLV (maximum curvature of the lateral ventricles [LVs]) and the CA (angle between the roof of the LVs) on the PC-level coronal plane; and the EI-z as the normalized MFH height (MFH-z/Max-z) on a range of coronal slices defined by the AC level.

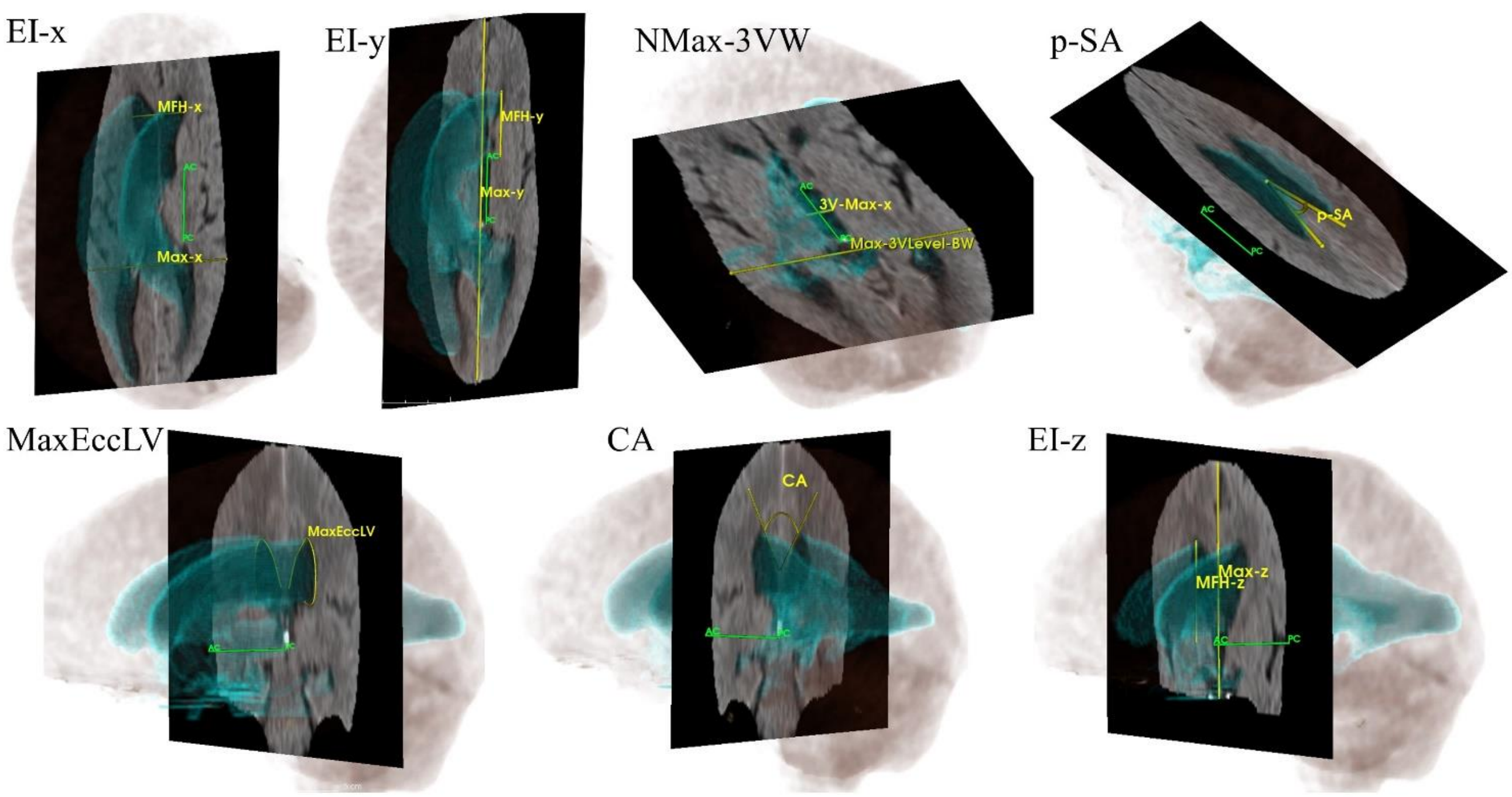

Supplemental Note 3. Impact of Precise AC-PC Localization on NPH Classification

To better quantify the impact of precise AC-PC localization on NPH classification, we applied our previously developed methodology (Kadaba Sridhar et al., 2024b) to compute the seven ventriculomegaly features (visualized in **Figure S3**) in reference to randomly perturbed reference standard AC-PC coordinates (hereafter, Features-PerturbACPC). Features-PerturbACPC were applied to train and test multivariate logistic regression (LR) models, under a 5-fold CV setup consistent with the AC-PC prediction evaluation (**Figure S4**), for classification of NPH from AD/PTVL/headache patients in the VA-Cohort-CS, under gradually increasing AC-PC localization errors induced by the random perturbations. These perturbations were equally introduced to each coordinate (x, y, and z) of the AC and the PC by adding random noise sampled from independent normal distributions with zero mean and gradually increasing standard deviations (1, 2, 3, 4, and 5 mm), which corresponded to MREs of 1.6, 3.2, 4.8, 6.4, and 8 mm (Euclidean distance between the perturbed and reference-standard

AC/PC landmarks). Classification scenarios examined were NPH vs headache patients, NPH vs AD, NPH vs PTVL, and NPH vs all. One-sample t-tests evaluated the differences between the mean of test-set AUCs from classification using 10 sets of Features-PerturbACPC assessments per noise level, and the test-set AUC from classifying with features assessed using the reference standard AC-PC (Features-RefACPC). The Benjamini-Hochberg (BH) method was used to adjust p-values (q-values), reducing the risk of false discovery in this multiple testing scenario (4 classification scenarios × 5 noise levels = 20 tests). Noise levels that started to induce a statistically significant ($q < 0.05$) and meaningful drop in AUC (> 0.01) were noted.

Figure S4. End-to-End Evaluation using 5-fold CV. Illustration of consistent train-test splits utilized to evaluate the AC-PC prediction framework (VA-Cohort) and the subsequent NPH classification (VA-Cohort-CS [VA-Cohort's chart-review qualified subset]) using ventriculomegaly features standardized with the our framework's AC-PC predictions (Features-PredACPC), the manual reference standard AC-PC (Features-RefACPC), and the perturbed AC-PC reference standard (Features-PerturbACPC). Distribution of the VA-Cohort-CS patients in the Registration-guided 3D-UNet evaluation (*green* dots representing AC-PC predictions) corresponded to that in the NPH Classification Evaluation (*green* triangles representing ventriculomegaly features) across the five cross-validation (CV) folds. VA = Veterans Affairs, NPH = Normal Pressure Hydrocephalus, AC = Anterior Commissure, PC = Posterior Commissure.

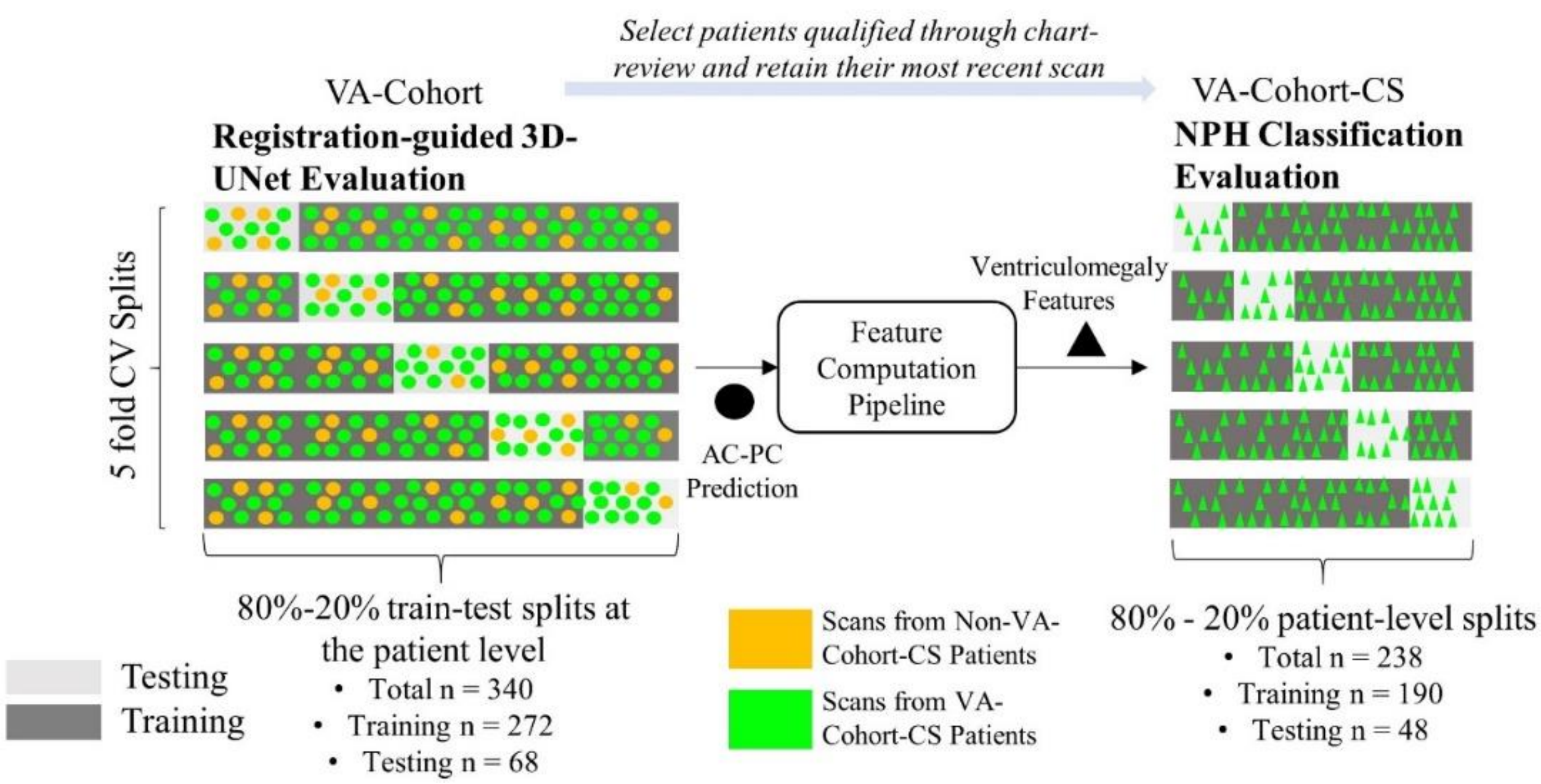

Table S3. Inter-Annotator Agreement in the VA-Cohort. Measures are on the VA-Cohort, between PA and SA-specific subsets. RE = Radial Error (in mm), AC = Anterior Commissure, PC = Posterior Commissure, PA = Primary Annotator, and SA = Secondary Annotator. Note that 36/427 scans, annotated in the initial round and subsequently used for analysis of multi-annotator agreement, are excluded here.

	**PA-SA1**	**PA-SA2**	**PA-SA3**	**PA-SA1, SA2,**
Number of scans	71	159	161	391
Mean ± Standard<br>AC RE<br>PC RE	 1.04 ± 0.58<br>1.09 ± 0.43	 0.95 ± 0.51<br>0.94 ± 0.46	 0.96 ± 0.55<br>0.91 ± 0.4	 0.97 ± 0.54<br>0.95 ± 0.44
75th percentile<br>AC RE<br>PC RE	 1.28<br>1.43	 1.17<br>1.2	 1.21<br>1.2	 1.22<br>1.25
Maximum<br>AC RE<br>PC RE	 3.25<br>2.37	 3.84<br>3.35	 3.49<br>2.04	 3.84<br>3.35
% scans within AC RE of<br>1.6 mm<br>3.2 mm<br>4.8 mm	 88.7%<br>98.6%<br>100.0%	 91.2%<br>98.7%<br>100.0%	 91.9%<br>98.1%<br>100.0%	 91%<br>98.5%<br>100.0%
% scans within PC RE of<br>1.6 mm<br>3.2 mm<br>4.8 mm	 83.1%<br>100.0%<br>100.0%	 95%<br>99.4%<br>100.0%	 93.8%<br>100.0%<br>100.0%	 92.3%<br>99.7%<br>100.0%

Figure S5. Multi-Annotator Agreement in the VA-Cohort. Multi-annotator agreement visualized using Extended Bland-Altman plots for each coordinate of the AC/PC annotations on a common set of 36 scans from the VA-Cohort. The annotator responsible for maximum deviation on each scan is indicated by the marker color, and annotator-specific biases are indicated by colored short ticks on the right. The limits of agreement (LOA) with 95% confidence intervals were within 1.1 mm for all AC/PC coordinates. Heterogeneity in marker colors suggested no annotator-specific biases. PA = Primary Annotator, SA = Secondary Annotator, AC = Anterior Commissure, and PC = Posterior Commissure. Note that the y and z coordinates of the AC/PC (x-axes in the plots) cluster in two distinct regions, reflecting the natural distribution of the physical origin in these scans during acquisition which also happened to cluster in two distinct regions.

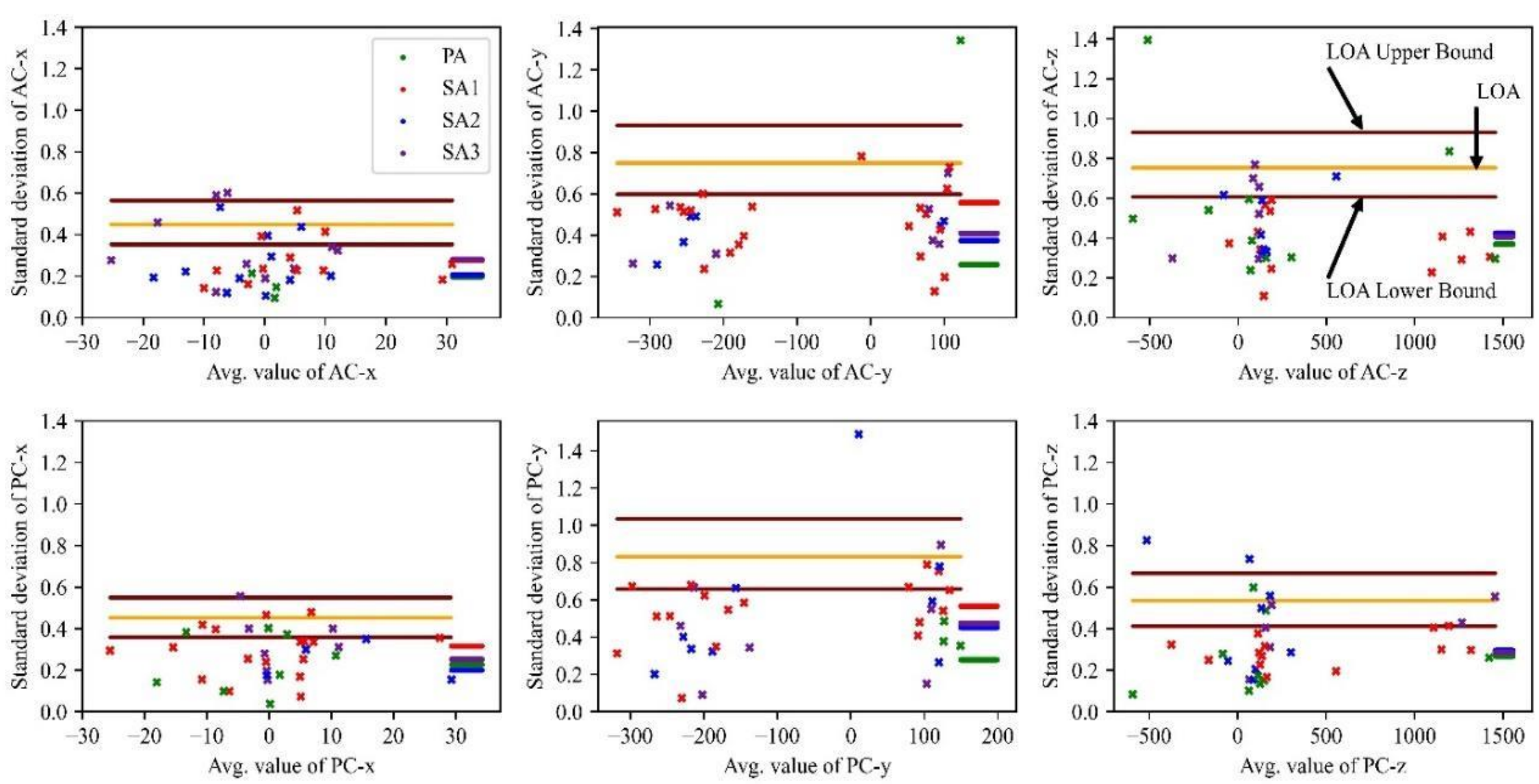

Table S4. Integrated vs. Reformat-Specific Baseline-, Coarse-, and Fine-Localization (registration-guided 3D-UNet framework). RE measures are indicated for the multi-planar VA-Cohort and the VA-ExtCohort. Note: The coronal baseline's artificially low errors on the VA-Cohort resulted from overfitting due to selecting hyperparameters based on test fold performance in a non-nested 5-fold split (See Section 2.7.1 in the main article for details). This lacks the rigorous, unbiased nested cross-validation evaluation of the registration-guided 3D-UNets. Furthermore, this baseline still underperformed coarse localization on the VA-Cohort and failed to generalize, yielding the highest PC error across the three reformats during external validation (VA-ExtCohort). MRE = Mean Radial Error, SD = Standard Deviation, prcntl. = percentile, Max. = Maximum, AC = Anterior Commissure, PC = Poster Commissure.

	AC RE	PC RE	AC RE	PC RE	AC RE	PC RE
	Mean ± SD	Mean ± SD	75th prcntl.	75th prcntl.	Max.	Max.
**VA-Cohort**						
Baseline Integrated	9.46±4.2	9.95±2.68	12.26	12.04	23.44	18.59
Baseline Axial	10.13 ± 5.18	10.97 ± 3.38	13.71	12.86	29.86	24.45
Baseline Coronal	5.14±2.67	3.37±1.75	6.64	4.42	14.36	10.88
Baseline Sagittal	12.08±2.63	11.71±2.24	13.74	13.26	19.77	18.28
Coarse Integrated	3.3±1.51	2.12±1.03	4.11	2.64	9.68	6.75
Coarse Axial	3.92 ± 1.69	2.37 ± 1.29	4.88	3.02	10.3	9.37
Coarse Coronal	3.6 ± 1.63	2.55 ± 1.23	4.38	3.21	9.5	6.88
Coarse Sagittal	3.56 ± 1.56	2.62 ± 1.29	4.34	3.24	10.63	10.17
Fine Integrated	1.64±0.83	1.49±0.72	2.00	1.87	5.85	4.07
Fine Axial	1.79±0.95	1.58±0.75	2.17	1.99	6.98	4.89
Fine Coronal	1.77±0.89	1.72±0.78	2.24	2.18	6.19	6.55
Fine Sagittal	2.12±0.99	2.04±1.01	2.64	2.61	6.33	7.61
**VA-ExtCohort**						
Baseline Integrated	13.56±5.64	15.52±3.99	14.9	18.07	33.67	26.23
Baseline Axial	14.63±9.29	17.55±7.32	15.39	21.08	51.16	45.13
Baseline Coronal	6.95±4.41	22.6±2.71	8.71	24.2	25.92	28.26
Baseline Sagittal	13.81±5.06	15.42±2.79	15.91	17.39	31.4	22.62
Coarse Integrated	3.9±1.99	2.97±1.6	5.08	4.08	10.04	8.8
Coarse Axial	4.39±2.27	3.2±2.0	5.05	3.95	11.38	11.3
Coarse Coronal	4.3±2.02	3.29±1.8	5.6	4.6	9.68	8.78
Coarse Sagittal	3.95±2.12	3.25±1.5	4.97	4.52	8.95	6.01
Fine Integrated	2.42 + 1.06	1.79 + 0.86	2.85	1.99	4.76	4.42
Fine Axial	2.41±0.97	1.7±1.04	2.79	2.07	5.43	5.42
Fine Coronal	2.74±1.28	2.11±1.09	3.37	2.49	6.47	5.32
Fine Sagittal	3.02±1.42	2.23±0.89	4.01	2.77	6.44	4.5

Figure S6. **Coordinate Errors in Integrated vs. Reformat-Specific Fine-Localization.** Absolute x/y/z coordinate errors of AC-PC predictions on the VA-Cohort's test sets and the VA-ExtCohort are indicated for both the reformat-specific and integrated predictions from the registration-guided 3D-UNet framework. Each coordinate's integrated prediction and the reformat-specific predictions averaged to generate it are shown in the same color. The excluded reformat is depicted in *orange*. Asterisks indicate statistical significance (Wilcoxon tests, Benjamini-Hochberg corrected significance level [q]) evaluating whether the absolute error of the integrated version was lower than that of the other reformats scan-wise. *: q < 0.05, **: q < 0.01, ***: q < 0.001, ns: not significant.

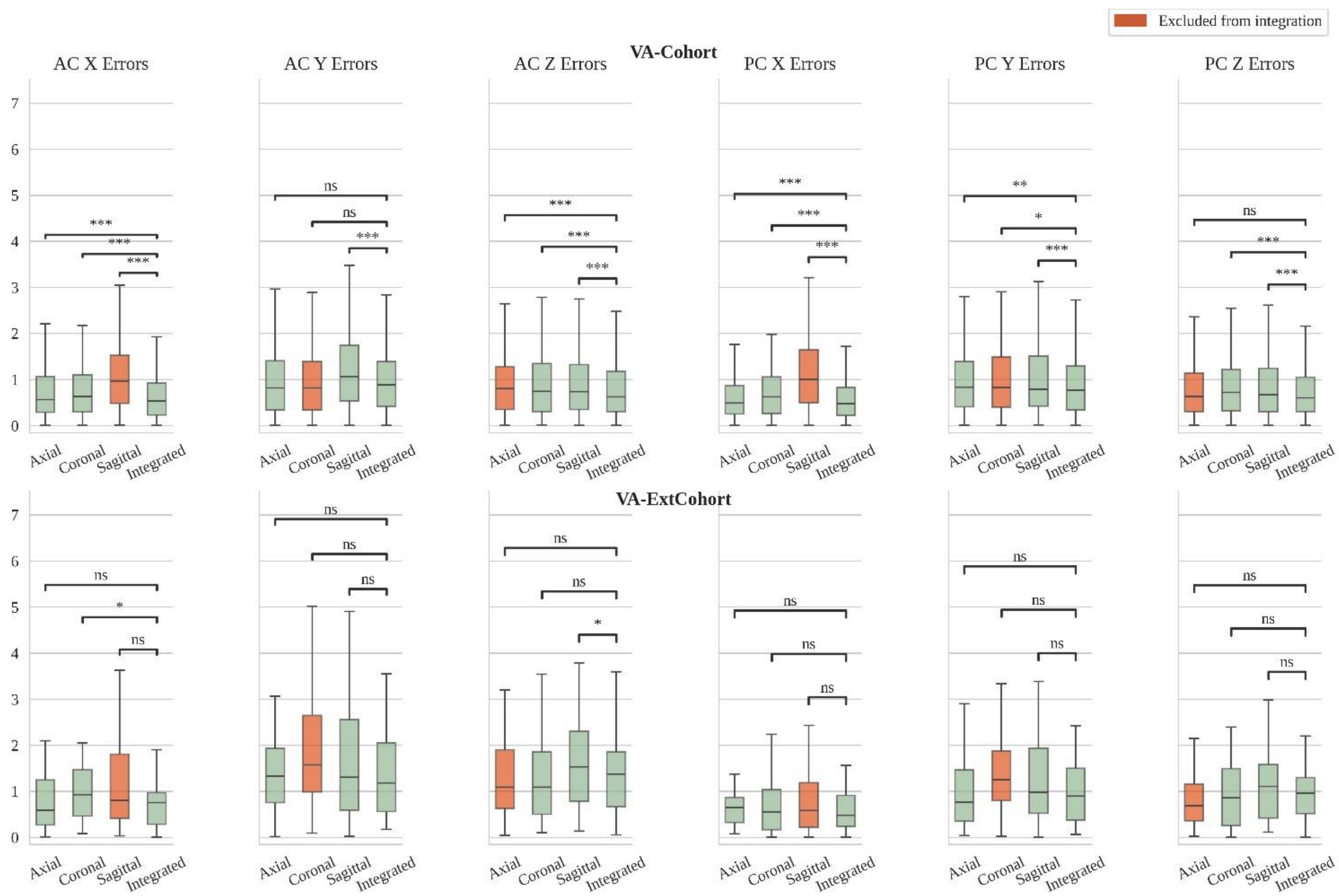

Table S5. Selected Hyperparameters (HPs). For each reformat-specific registration-guided 3D-UNet, twenty-five sets of HPs were evaluated using random search. The learning rate (lr) was sampled from a log-uniform distribution between 10^{-4} and 10^{-1}. The class-imbalance parameter (α) ranged from 0.1 to 0.4 (step size 0.1), the focus parameter (γ) from 0 to 5 (step size 1), and the Gaussian heatmap size (σ) from 4 to 14 (step size 2). HP combinations were evaluated via nested 5-fold cross-validation (CV), and the combination with the lowest overall mean radial error (MRE = [AC MRE + PC MRE]/2) across the inner CV folds was selected for training the final models (**indicated in this table**), followed by evaluation on the held-out outer test-folds. HPs for the reformat-specific baseline models were evaluated with the same sampling strategy as the registration-guided 3D-UNets, under 5-fold CV. The combination with the lowest MRE across the test-folds was chosen as the final version. AC = Anterior Commissure, PC = Posterior Commissure.

	Registration-guided 3D-UNets				Baseline 3D-UNets			
Reformat	lr	α	γ	σ	lr	α	γ	σ
Axial	0.0563	0.8	1	4	0.0001	0.6	5	14
Coronal	0.0716	0.7	3	4	0.0006	0.6	0	14
Sagittal	0.0229	0.8	1	6	0.0006	0.7	0	8

Figure S7. Training and validation loss trajectories. For each reformat-specific model (Axial, Coronal, and Sagittal), these curves represent the learning dynamics from the nested cross-validation (hyperparameter tuning) phase. Specifically, the plots display the mean loss and ±1 standard deviation shaded variance across all 20 inner-loop validation runs (5 outer folds × 4 inner folds) for the final selected hyperparameter configuration. Note that the y-axes are presented on a logarithmic scale. All three models exhibit a rapid initial decrease in both training and validation loss, followed by a distinct inflection point (~ epoch 8) after which the loss plateaus. Crucially, across the remaining epochs up to epoch 25, the validation loss remains stable and flat. No upward trajectory in the validation loss curves is observed, confirming no overfitting.

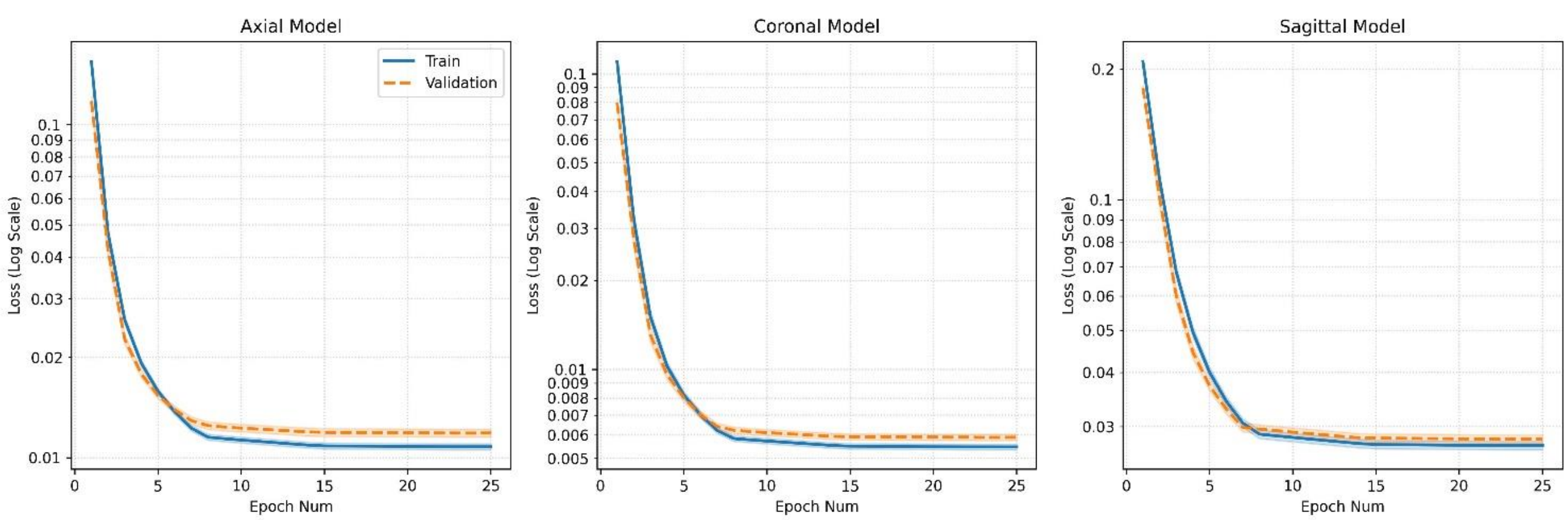

Table S6. Predicted/True Class Frequencies in NPH Classification. Counts corresponding to Table 5 showing classification performance of ventriculomegaly features computed using Predicted (P) vs Reference Standard (R) AC-PC Locations, on the VA-Cohort-CS dataset. NPH = Normal Pressure Hydrocephalus, AD = Alzheimer's disease, PTVL = Post-traumatic volume loss, Acc = Accuracy, Prec = Precision, Sens = Sensitivity, Spec = Specificity, N/D = Numerator/Denominator. P/R denominators for accuracy/sensitivity/specificity remain the same as they depend on true class labels (NPH/AD/PTVL/headache patients). Numbers are reported by accumulating class predictions over held-out test-folds.

NPH vs.	Acc N	Acc D	Prec N	Prec D	Sens N	Sens D	Spec N	Spec D
	P|R		P|R	P|R	P|R		P|R	
AD	107 | 108	126	53 | 54	61 | 62	53 | 54	64	54 | 54	62
PTVL	106 | 104	117	60 | 58	67 | 65	60 | 58	64	46 | 46	53
Headache	122 | 122	123	64 | 64	65 | 65	64 | 64	64	58 | 58	59
All	211 | 207	238	59 | 58	81 | 83	59 | 58	64	152 | 149	174

Table S7. Ventriculomegaly Features Using Predicted vs. Reference Standard AC-PC. Mean signed differences (d) between ventriculomegaly features assessed using reference standard AC-PC (Features-RefACPC) vs predicted AC-PC (Features-PredACPC) landmarks on the VA-Cohort-CS dataset, evaluated via paired t-tests. Benjamini-Hochberg corrected significance level (q) and 95% confidence intervals (CI) are given. EI = Evans Index, NMax-3VW = Normalized 3rd Ventricle Width, MaxEccLV = Maximum Eccentricity of the Lateral Ventricles, CA = Callosal Angle, p-SA = proxy-Splenial Angle, AC = Anterior Commissure, PC = Posterior Commissure. CA and p-SA are in degrees.

Feature	Features-	Features-	d	q	95% CI
EI-x	0.3148	0.3153	-0.0005	0.3	[-0.001,0.0]
EI-y	0.1828	0.1858	-0.003	<.001	[-0.004,-0.002]
EI-z	0.3129	0.3157	-0.0028	0.01	[-0.005,-0.001]
NMax-3VW	0.0547	0.0548	-0.0002	0.45	[-0.001,0.0]
MaxEccLV	0.8716	0.8685	0.003	0.14	[-0.001,0.007]
CA	102.1897	100.7425	1.4473	0.06	[0.186,2.708]
p-SA	53.2599	52.1224	1.1374	0.12	[-0.099,2.374]

Table S8. Feature-specific NPH Classification. Feature-wise AUC for classification of NPH from AD/PTVL/headache patients using logistic regression (LR), by assessing the ventriculomegaly features in reference to predicted (P) versus reference standard AC-PC (R), on the VA-Cohort-CS dataset. “All” refers to a multivariate LR with all the seven features. Evaluation is under the 5-fold CV described in Figure S3. EI = Evans Index, NMax-3VW = Normalized 3rd Ventricle Width, MaxEccLV = Maximum Eccentricity of the Lateral Ventricles, CA = Callosal Angle, p-SA = proxy-Splenial Angle, AC = Anterior Commissure, PC = Posterior Commissure, NPH = Normal Pressure Hydrocephalus, AD = Alzheimer’s disease, PTVL = Post-traumatic volume loss.

NPH vs.	All	EI-x	EI-y	EI-z	NMax-3VW	MaxEcc-LV	p-SA	CA
	P|R	P|R	P|R	P|R	P|R	P|R	P|R	P|R
AD	0.92|0.93	0.82|0.81	0.73|0.76	0.91|0.91	0.82|0.81	0.87|0.85	0.63|0.55	0.8|0.81
PTVL	0.94|0.94	0.87|0.87	0.78|0.75	0.95|0.96	0.85|0.86	0.9|0.93	0.64|0.66	0.84|0.86
Headache patients	0.98|0.98	0.98|0.98	0.93|0.84	0.98|0.98	0.97|0.98	0.93|0.96	0.54|0.64	0.85|0.86
All	0.95|0.95	0.89|0.89	0.83|0.8	0.95|0.96	0.89|0.89	0.91|0.92	0.59|0.63	0.82|0.85
